# Biodegradable electrospun nanofibrous platform integrating antiplatelet therapy-chemotherapy for preventing postoperative tumor recurrence and metastasis

**DOI:** 10.7150/thno.69795

**Published:** 2022-04-24

**Authors:** Jianye Li, Jiaojiao Li, Yuzhu Yao, Tuying Yong, Nana Bie, Zhaohan Wei, Xin Li, Shiyu Li, Jiaqi Qin, Haibo Jia, Qing Du, Xiangliang Yang, Lu Gan

**Affiliations:** 1 National Engineering Research Center for Nanomedicine, College of Life Science and Technology, Huazhong University of Science and Technology, Wuhan 430074, China.; 2 Key Laboratory of Molecular Biophysics of the Ministry of Education, College of Life Science and Technology, Huazhong University of Science and Technology, Wuhan 430074, China.; 3 Hubei Key Laboratory of Bioinorganic Chemistry and Materia Medica, Huazhong University of Science and Technology, Wuhan 430074, China.; 4 Hubei Engineering Research Center for Biomaterials and Medical Protective Materials, Huazhong University of Science and Technology, Wuhan 430074, China.

**Keywords:** Antiplatelet therapy, Doxorubicin-loaded tumor repopulating cell-derived microparticles, Electrospun nanofibrous films, *In situ* implantation, Tumor recurrence and metastasis

## Abstract

The perioperative trauma-related platelet recruitment and activation severely affect tumor recurrence and metastasis. Therefore, efficiently killing residual tumor cells and simultaneously inhibiting platelet activation to block platelet-cancer cell interaction might be a promising strategy to prevent postoperative tumor recurrence and metastasis.

**Methods:** Biodegradable PLGA electrospun nanofibrous films co-delivering doxorubicin-loaded tumor repopulating cell-derived microparticles (DOX-MPs) and aspirin (ASA) were developed as the implant materials (DOX-MPs/ASA@NF) for postoperative in-situ treatment. The characterization, cytotoxicity against tumor cells, inhibition in platelet activation-triggered proliferation, migration and metastasis of tumor cells and *in vivo* anti-recurrence and anti-metastasis activity induced by DOX-MPs/ASA@NF were systematically evaluated.

**Results:** PLGA nanofibrous films facilitate the enhanced distribution of DOX-MPs as well as DOX-MPs and ASA release in a time-programmed manner within the tumor resection cavity. The released DOX-MPs efficiently kill the residual tumor cells, while ASA decreases platelet activation and inhibits platelet-promoted proliferation, migration and metastasis of tumor cells, resulting in the remarkable inhibition of postoperative tumor recurrence and metastasis.

**Conclusions:** DOX-MPs/ASA@NF may be a promising candidate to prevent the recurrence and metastasis of resectable tumors.

## Introduction

Surgical resection is the primary treatment modality for most solid tumors 1,2. However, the curative effects of surgical resection are limited by the subsequent relapse and metastasis from residual tumor cells, leading to significant morbidity and mortality 3-5. Adjuvant therapies, including chemotherapy and radiotherapy are important options for eradicating residual tumor cells following surgical resection, but these treatments often cause severe side effects, leading to limited efficacy in improving the overall survival 2,6,7. Therefore, it is urgently needed to develop an appropriate postoperative therapeutic strategy to kill residual tumor cells to prevent tumor recurrence and metastasis.

Besides residual tumor cells, the perioperative trauma-related tumor microenvironment severely affects the tumor recurrence and metastasis 5,8. Platelets, one of the vital components in the blood circulation, play a key role in hemostasis 9-11. As a guard, the platelets rapidly detect and are recruited to the disruption site of the abnormal blood vessel injury to stop the bleeding and help repair the injury 6,9. Mounting evidences show that activated platelets can promote the proliferation, epithelial-mesenchymal transition (EMT) and metastasis of tumor cells by releasing a variety of cytokines and chemokines such as transforming growth factor-beta (TGF-β) 12-14. In contrast, tumor cells also induce platelet activation 15,16. The cycle of interaction between activated platelets and tumor cells results in an increased tumor recurrence and metastasis. Therefore, efficiently killing residual tumor cells and simultaneously inhibiting platelet activation to block platelet-cancer cell interaction might be a promising strategy to prevent postoperative tumor recurrence and metastasis. Aspirin (also known as acetylsalicylic acid, ASA), the well-known nonsteroidal anti-inflammatory drug, is one of the most widely used antiplatelet medications 17-19. ASA is an irreversible cyclooxygenase (COX) inhibitor through acetylation of a serine residue that reduces the synthesis of prostanoids, such as prostaglandin E2 (PGE2) and thromboxane A2 (TXA2) 20,21. Recent works showed that aspirin administration reduced distant metastases in murine models and clinical trials due to inhibition of COX-1/TXA2 pathway in platelets, resulting in the decreased aggregation of platelets on tumor cells and diminished formation of a premetastatic niche 20-22. However, systemic administration of ASA brings risk of severe bleeding complication.

**Figure 1:**
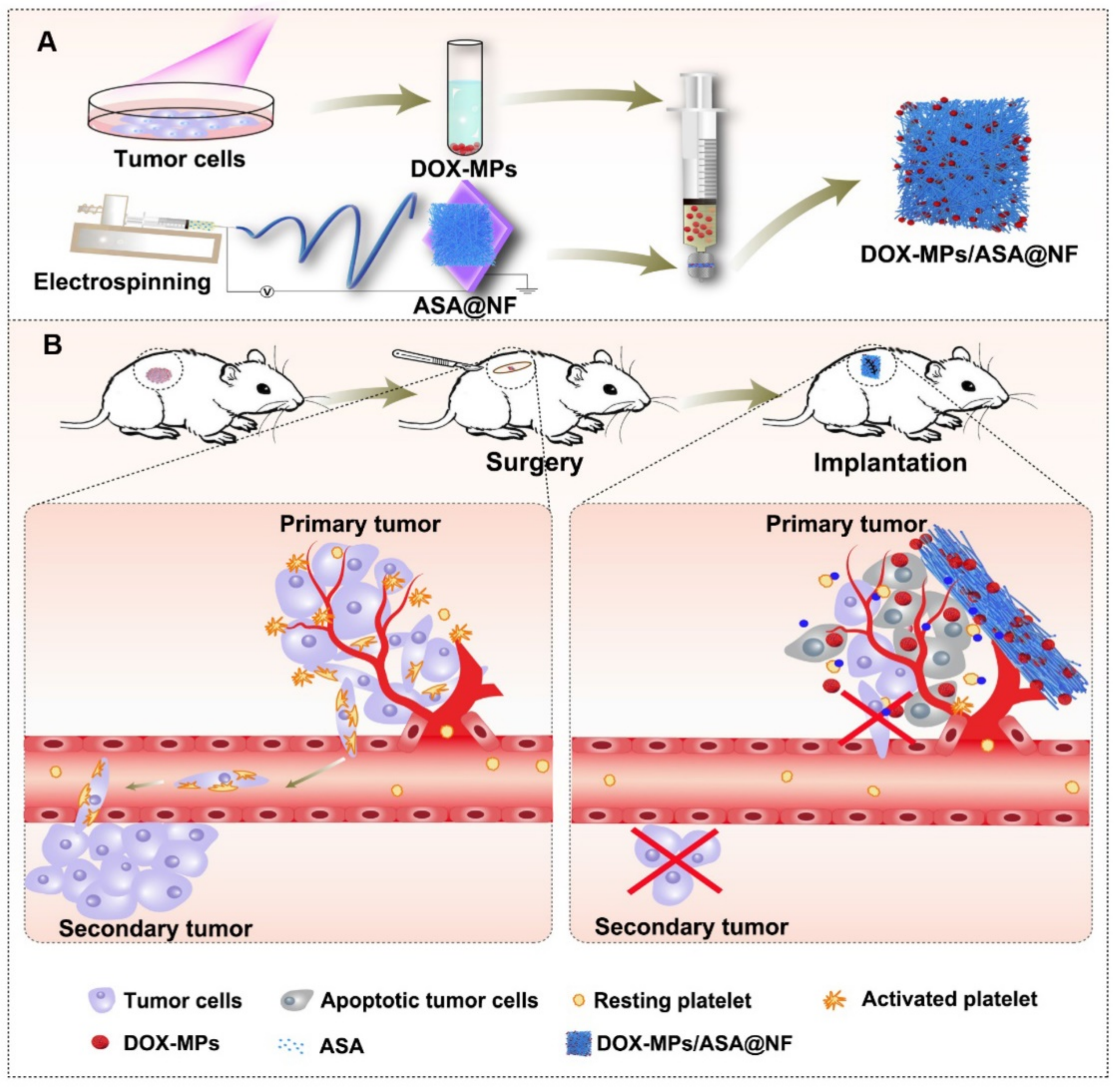
** Schematic illustration of DOX-MPs/ASA@NF for postoperative in-situ treatment. (A)** Schematic illustration of the preparation of DOX-MPs/ASA@NF. **(B)** Schematic illustration of DOX-MPs/ASA@NF for inhibiting postoperative tumor recurrence and metastasis. DOX-MPs/ASA@NF exhibits sustained and time-programmed release of DOX-MPs and ASA in the tumor resection cavity. The fast release of DOX-MPs efficiently kills residual tumor cells, while the slow ASA release decreases platelet activation to inhibit the platelet-promoted proliferation, migration and metastasis of tumor cells.

Drug-loaded implants can be applied directly to the resection site and offer enhanced drug accumulation at the targeted site, localized and prolonged drug release with minimal side effects 23, providing a promising strategy for postoperative cancer treatment. Among them, the implants composed of electrospun nanofibrous (NF) films attract great attention as drug delivery carriers due to the large surface area-to-volume ratio, uniformity in fiber size, high porosity and diversity of components 24,25. Extracellular vesicles, including microparticles (MPs) and exosomes with high biocompatibility, intrinsic targeting properties and low immunogenicity are used as ideal anticancer drug delivery carriers 26-28. Recently, our group developed tumor-repopulating cell (TRC)-derived microparticles (MPs) loading anticancer drug to efficiently inhibit tumor growth 26. The anticancer drug can be efficiently targeted to tumor tissues and internalized into tumor cells owing to the unique softness of these MPs 26. Here, we construct a biocompatible poly(DL-lactide-co-glycolide) (PLGA) electrospun NF films co-delivering doxorubicin (DOX)-loaded TRC-derived MPs (denoted as DOX-MPs) and ASA as the implant materials (denoted as DOX-MPs/ASA@NF, Figure 1A) for postoperative in-situ treatment. DOX-MPs/ASA@NF sustainedly and time-programmedly releases DOX-MPs and ASA in the surgical tumor cavity, in which more DOX-MPs can be accumulated in tumor tissues and efficiently kill residual tumor cells, while ASA decreases platelet activation to inhibit the platelet-promoted proliferation, migration and metastasis of tumor cells. Combined with these characters, DOX-MPs/ASA@NF efficiently inhibits postoperative tumor recurrence and metastasis (Figure 1B).

## Results and Discussion

### Preparation and characterization of DOX-MPs/ASA@NF

DOX-MPs/ASA@NF was constructed as the implant materials for postsurgical in-situ treatment (Figure 1A). The PLGA NF films (denoted as NF) delivering ASA (denoted as ASA@NF) were first fabricated by electrospinning using the mixture of PLGA and ASA as the spinning solution, which was confirmed by Fourier transform infrared spectroscopy (Figure S1). DOX-MPs were collected by treating murine hepatocellular carcinoma H22 TRCs or breast cancer 4T1 TRCs with DOX, followed by ultraviolet irradiation for MP induction. Dynamic light scattering (DLS) analysis revealed that DOX-MPs had an average diameter of 417 nm (Figure S2A) and zeta potential of -16.1 mV. DOX-MPs were monodisperse and irregularly spherical observed by transmission electron microscope (TEM, Figure S2B). The encapsulation efficacy of MPs for DOX was 1.27 ± 0.13%. Furthermore, ASA@NF was put into the filter as a filter membrane and DOX-MPs solution was then pushed through the filter using injection syringe to prepare DOX-MPs/ASA@NF.

**Figure 2:**
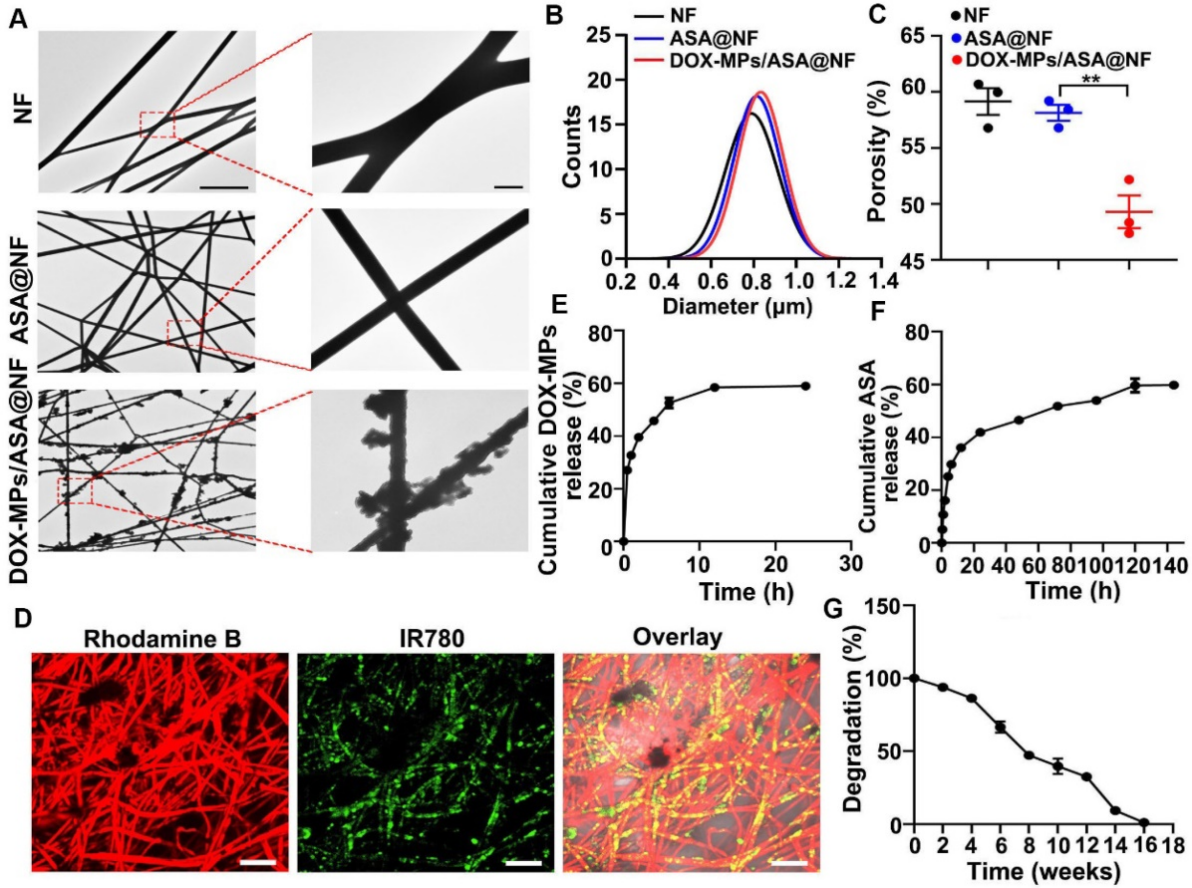
** Characterization of DOX-MPs/ASA@NF. (A)** TEM images of NF, ASA@NF and DOX-MPs/ASA@NF. The right images were the amplification of the insets in the left images. Scale bars: 10 µm (left) and 1 µm (right). **(B)** The diameter of NF, ASA@NF and DOX-MPs/ASA@NF by SEM analysis. **(C)** The porosity of NF, ASA@NF and DOX-MPs/ASA@NF by liquid intrusion method. **(D)** Confocal microscopic images of IR780-MPs/Rhodamine@NF. Scale bars: 10 µm. **(E,F)** DOX-MPs (E) and ASA (F) release profiles from DOX-MPs/ASA@NF in PBS at pH 7.4. **(G)** *In vitro* degradation profiles of PLGA NF films in PBS with 8 µg mL^-1^ proteinase K and 4 mg mL^-1^ lysozyme. Data are presented as mean ± s.e.m. (n = 3). **P < 0.01.

NF and ASA@NF had smooth surface by TEM (Figure 2A) and scanning electronic microscope (SEM, Figure S3), while DOX-MPs/ASA@NF exhibited rough surface (Figure 2A and Figure S3), suggesting that DOX-MPs might absorb on the NF film surface and the interspaces between NF films. Colocalization of DOX fluorescence with ASA@NF in DOX-MPs/ASA@NF revealed that DOX-MPs were loaded into ASA@NF (Figure S4). The average diameter of NF, ASA@NF and DOX-MPs/ASA@NF was about 790 nm, 814 nm and 833 nm measured by SEM, respectively (Figure 2B). The porosity of NF, ASA@NF and DOX-MPs/ASA@NF was about 59.1%, 58.1% and 49.3% estimated by liquid intrusion method, respectively (Figure 2C). The decreased porosity of DOX-MPs/ASA@NF confirmed that DOX-MPs were loaded into multiple gaps in NF films. Rhodamine B (a model drug replacing ASA) and IR780 (a model drug replacing DOX)-MPs were clearly observed in IR780-MPs/Rhodamine B@NF by confocal microscopy (Figure 2D), confirming that ASA and DOX-MPs can be efficiently loaded into NF films. The encapsulation efficacy of NF films was 92.7% and 54.7% for ASA and DOX-MPs, respectively. The drug loading efficiency of ASA and DOX-MPs in DOX-MPs/ASA@NF was 92.7 μg mg^-1^ NF and 1.2 μg mg^-1^ NF, respectively. The drug release analysis showed that DOX-MPs and ASA exhibited a sustained drug release kinetics, in which DOX-MPs release reached about 58.9% during 24 h (Figure 2E) while ASA release reached about 59.6% until 120 h (Figure 2F). The faster DOX-MPs release and slower ASA release contributed to the efficient killing of tumor cells by DOX-MPs and the lasting deactivation of platelets to inhibit tumor recurrence and metastasis. The *in vitro* degradation curves showed that the PLGA NF films were degraded slowly and became undetectable within 16 weeks in PBS with proteinase K and lysozyme (Figure 2G), which can degrade PLGA in tumor microenvironment 2. Meanwhile, the *in vivo* degradation analysis revealed that PLGA NF films were completely degraded during 12 weeks after subcutaneous implantation into the flanks of BALB/c mice (Figure S5). In addition, hematoxylin and eosin (H&E) staining showed that no significant toxicity was observed in the surrounding skins and abdominal muscle tissues after implantation of PLGA NF films (Figure S6). These data suggested that the PLGA NF films were biodegradable and biocompatible. In addition, PLGA NF films exhibited blood-clotting ability similar to commercially available gelatin sponge (Figure S7).

### DOX-MPs/ASA@NF efficiently induces cytotoxicity against tumor cells

**Figure 3:**
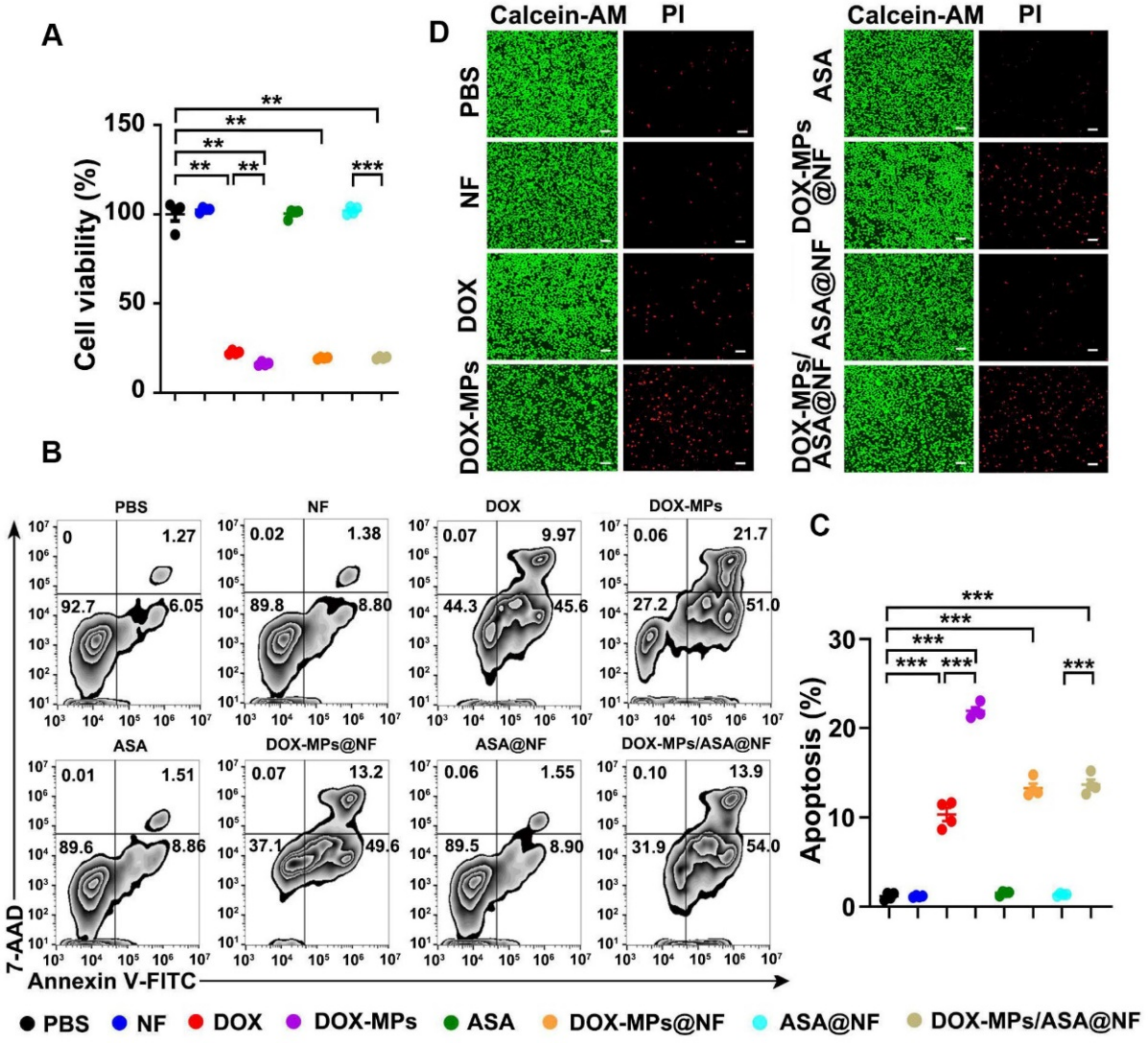
***In vitro* cytotoxicity of DOX-MPs/ASA@NF against 4T1 cells. (A)** Cell viability of 4T1 cells after treatment with PBS, NF, DOX, DOX-MPs, ASA, DOX-MPs@NF, ASA@NF or DOX-MPs/ASA@NF at the DOX concentration of 2 µg mL^-1^ and ASA concentration of 150 µg mL^-1^ for 24 h by CCK-8 assay. **(B,C)** Representative flow cytometric images (B) and the quantification (C) of apoptosis in 4T1 cells after treatment as above by Annexin V apoptosis kit with 7-AAD. Data are presented as mean ± s.e.m. (n = 4). *P < 0.05, **P < 0.01, ***P < 0.001. **(D)** Representative fluorescence microscopic images of 4T1 cells stained with calcein-AM/PI after treatment as above. Scale bars: 100 µm.

To determine the direct killing efficacy of DOX-MPs/ASA@NF against tumor cells, 4T1 or H22 cells were treated with PBS, NF, DOX, DOX-MPs, ASA, DOX-MPs@NF, ASA@NF or DOX-MPs/ASA@NF, and the cell viability was then determined by CCK-8 assay (Figure 3A and Figure S8A). As expected, NF, ASA and ASA@NF did not markedly affect the viability of 4T1 and H22 cells, suggesting that NF and ASA did not induce significant toxicity to tumor cells. Consistent with our previous work 26, DOX-MPs exhibited stronger cellular internalization (Figure S9) and cytotoxicity against 4T1 and H22 cells than DOX, which might be due to the homologous targeting and the unique softness of MPs. DOX-MPs@NF and DOX-MPs/ASA@NF showed similar cell viability with DOX-MPs, revealing that DOX-MPs/ASA@NF efficiently induced cytotoxicity against tumor cells. Furthermore, DOX-MPs/ASA@NF-induced apoptosis in 4T1 (Figure 3B-C) and H22 cells (Figure S8B-C) was evaluated using Annexin V apoptosis kit with 7-amino-actinomycin-D (7-AAD). Consistently, DOX-MPs induced stronger apoptosis in 4T1 and H22 cells than DOX. Loading ASA to NF did not significantly affect the DOX-MPs@NF-induced apoptosis. In addition, live/dead cell staining analysis showed that no significant red fluorescence of PI emitted by dead cells was observed in NF-, ASA- or ASA@NF-treated group compared with control group (Figure 3D). However, DOX-MPs-, DOX-MPs@NF- or DOX-MPs/ASA@NF-treated group exhibited stronger red fluorescence (Figure 3D), confirming that DOX-MPs/ASA@NF showed remarkable killing ability against tumor cells.

### DOX-MPs/ASA@NF efficiently inhibits platelet activation

**Figure 4:**
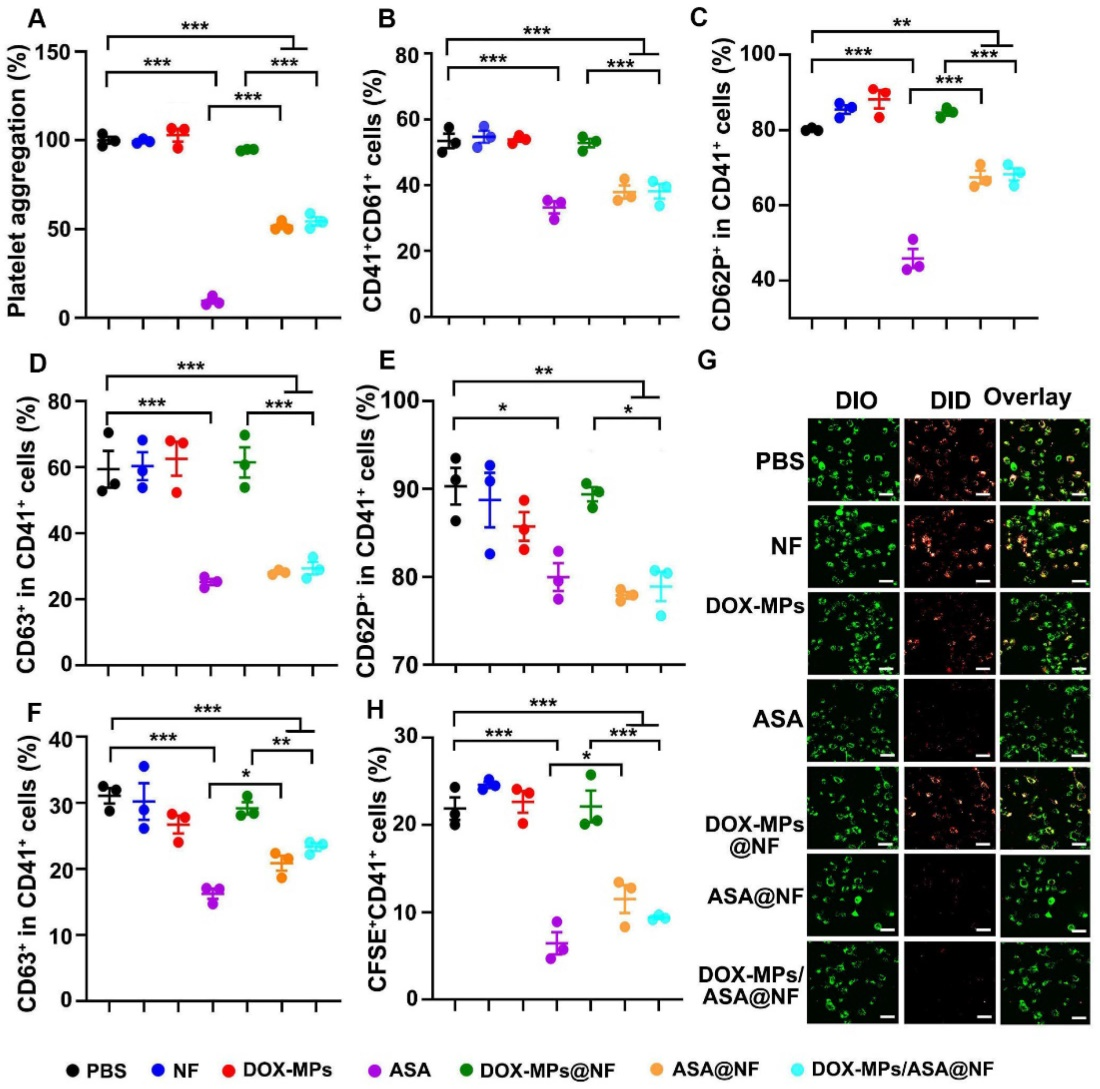
** Efficient inhibition of platelet activation induced by DOX-MPs/ASA@NF. (A)** Platelet aggregation after AA-activated PRP was treated with PBS, NF, DOX-MPs, ASA, DOX-MPs@NF, ASA@NF or DOX-MPs/ASA@NF at the DOX concentration of 8 µg mL^-1^ and ASA concentration of 600 µg mL^-1^ for 12 h. **(B)** Percentage of CD41^+^CD61^+^ cells after anti-CD41 or anti-CD61 antibody-labeled platelets pretreated as above were mixed in the presence of 0.5 mM AA for 15 min by flow cytometry. **(C,D)** Percentage of CD62P^+^ (C) and CD63^+^ cells (D) in CD41^+^ platelets after AA-activated platelets were treated as above by flow cytometry. **(E,F)** Percentage of CD62P^+^ (E) and CD63^+^ cells (F) in CD41^+^ platelets after 4T1 cell-activated platelets were treated as above by flow cytometry. **(G)** Confocal microscopic images of DIO-labeled 4T1 cells incubated with DID-labeled platelets pretreated as above for 8 h. Scale bars: 50 µm. **(H)** Percentage of CFSE^+^CD41^+^ cells after CFSE^+^-labeled 4T1 cells were incubated with anti-CD41 antibody-labeled platelets pretreated as above for 8 h by flow cytometry. Data are presented as mean ± s.e.m. (n = 3). *P < 0.05, **P < 0.01, ***P < 0.001.

Inhibition of platelet function was crucial for cutting off the interaction between the platelets and tumor cells 29-31. Aspirin represses platelet function through irreversible inhibition of cyclooxygenase (COX) activity 22,32,33. To determine whether DOX-MPs/ASA@NF suppressed platelet function, the effects of DOX-MPs/ASA@NF on arachidonic acid (AA)-activated platelet aggregation were first determined. The AA-activated platelets were treated with PBS, NF, DOX-MPs, ASA, DOX-MPs@NF, ASA@NF or DOX-MPs/ASA@NF, and the light transmittance of platelet suspensions after treatments was measured at 650 nm (Figure 4A). Compared with PBS, treatment with NF, DOX-MPs or DOX-MPs@NF did not significantly affect the platelet aggregation. In contrast, ASA, ASA@NF and DOX-MPs/ASA@NF exhibited significant inhibition in platelet aggregation. Furthermore, the above treated platelets were labeled with anti-CD41 or anti-CD61 antibody, respectively, and the CD41^+^CD61^+^ platelets were regarded as the aggregated platelets after mixing (Figure 4B). Consistently, compared with PBS-, NF-, DOX-MPs- or DOX-MPs@NF-treated group, the ratio of CD41^+^CD61^+^ platelets was significantly decreased in ASA-, ASA@NF- or DOX-MPs/ASA@NF-treated group, confirming that DOX-MPs/ASA@NF efficiently inhibited platelet aggregation. Besides platelet aggregation, surveillance of platelet granule secretion is an important and sensitive marker for platelet activation 34. The expression of CD62P and CD63 in platelets was used for monitoring the secretion of alpha granule and dense or lysosome granule, respectively 35. As expected, ASA, ASA@NF and DOX-MPs/ASA@NF significantly decreased the ratio of CD62P^+^CD41^+^ (Figure 4C and Figure S10) and CD63^+^CD41^+^ cells (Figure 4D and Figure S10) on the AA-activated platelets compared with PBS, NF, DOX-MPs and DOX-MPs@NF by flow cytometry, revealing the efficient inhibition of platelet activation by DOX-MPs/ASA@NF. The DOX-MPs/ASA@NF-induced decrease in the ratio of CD62P^+^CD41^+^ (Figure 4E) and CD63^+^CD41^+^ cells (Figure 4F) was further verified in tumor cell-activated platelets.

The binding of platelets to tumor cells, leading to platelet activation, plays a key role in the regulation of tumor growth and metastasis 36,37. To determine whether DOX-MPs/ASA@NF influenced the binding of platelets to tumor cells, the DID-labeled platelets treated with PBS, NF, DOX-MPs, ASA, DOX-MPs@NF, ASA@NF or DOX-MPs/ASA@NF were co-incubated with DIO-labeled 4T1 cells, followed by washing with PBS and then observing the adhesion of platelets to 4T1 cells using confocal microscopy (Figure 4G). Remarkable overlay of DID-labeled platelets and DIO-labeled 4T1 cells was observed in PBS-, NF-, DOX-MPs- or DOX-MPs@NF-treated group. However, treatment with ASA, ASA@NF or DOX-MPs/ASA@NF significantly decreased the adhesion of platelets to 4T1 cells. Moreover, when the above treated platelets labeled with anti-CD41-APC antibody were co-incubated with CFSE-labeled 4T1 cells, the ratio of CFSE^+^CD41^+^ cells in the ASA-, ASA@NF- or DOX-MPs/ASA@NF-treated group was significantly lower than other groups by flow cytometry (Figure 4H), further confirming that DOX-MPs/ASA@NF efficiently decreased the binding of platelets to tumor cells, resulting in the inhibition of platelet activation.

### DOX-MPs/ASA@NF-induced inhibition of platelet activation prevents the proliferation, migration and metastasis of tumor cells *in vitro*

**Figure 5:**
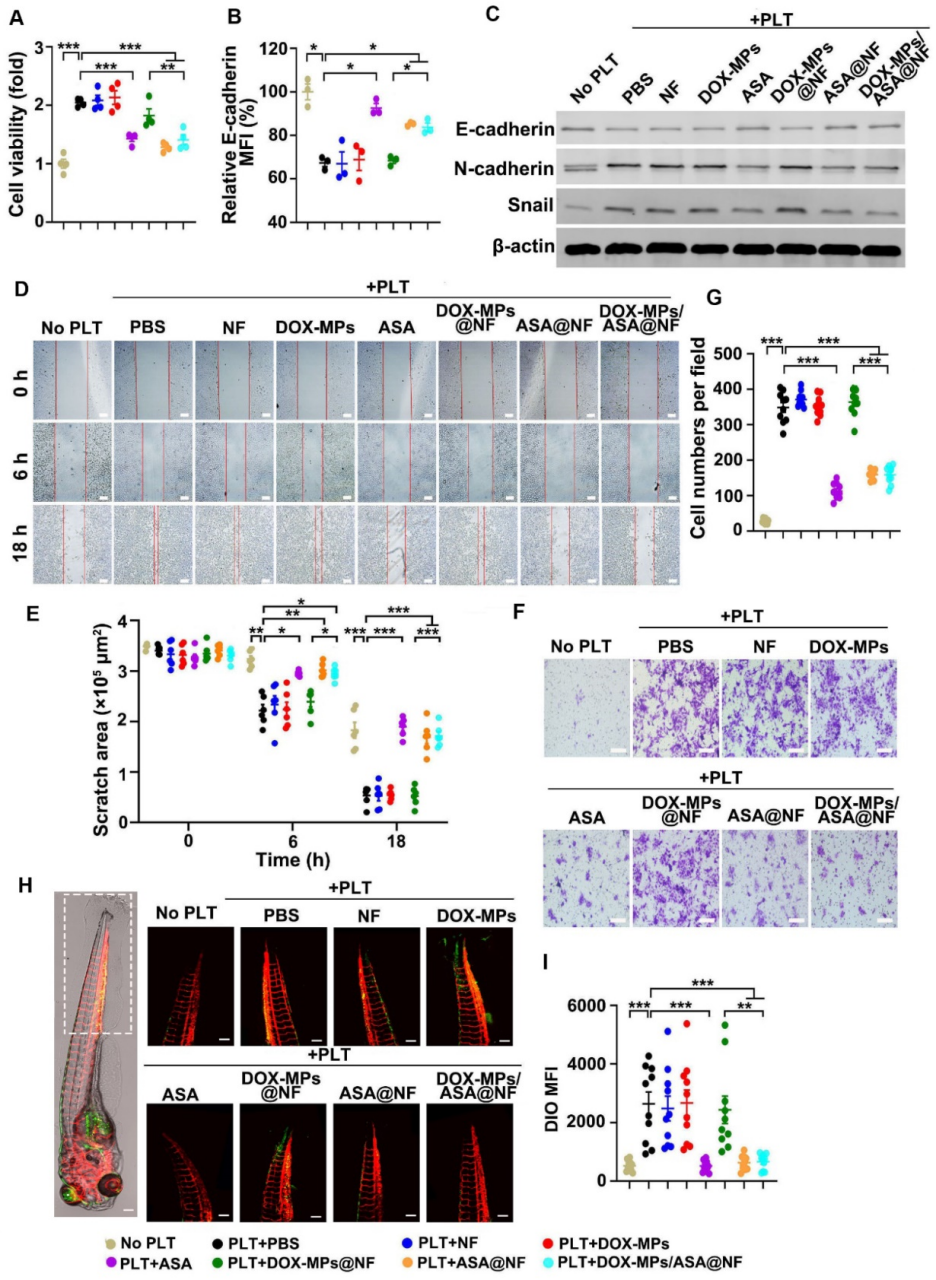
** Effects of DOX-MPs/ASA@NF-triggered platelet activation inhibition on the proliferation, migration and metastasis of 4T1 cells. (A)** Cell viability of 4T1 cells at 48 h after incubation with or without PBS-, NF-, DOX-MPs-, ASA-, DOX-MPs@NF-, ASA@NF- or DOX-MPs/ASA@NF-pretreated platelets at the DOX concentration of 8 μg mL^-1^ and ASA concentration of 600 µg mL^-1^ for 12 h. Data are presented as mean ± s.e.m. (n = 4). **(B)** E-cadherin expression in 4T1 cells at 24 h after incubation with or without the pretreated platelets as above by flow cytometry. Data are presented as mean ± s.e.m. (n = 3). *P < 0.05, **P < 0.01, ***P < 0.001. **(C)** E-cadherin, N-cadherin and Snail expression in 4T1 cells at 24 h after incubation with or without the pretreated platelets as above by western blot. **(D,E)** Representative images of scratch assay (D) and the quantification of scratch area (E) in 4T1 cells at the different time points after incubation with or without the pretreated platelets as above. Scale bars: 100 µm. Data are presented as mean ± s.e.m. (n = 6). *P < 0.05, **P < 0.01, ***P < 0.001. **(F,G)** Representative images (F) and the quantification (G) of migratory 4T1 cells stained with crystal violet at 12 h after incubation with or without the pretreated platelets as above by transwell assay. Scale bars: 50 µm. Data are presented as mean ± s.e.m. (n = 10). ***P < 0.001. **(H,I)** Representative images (H) and the quantification (I) of metastatic 4T1 cells in the tails of zebrafish embryos at 72 h after co-injection of DIO-labeled 4T1 cells (green) and the pretreated platelets as above. The area showing the metastatic 4T1 cells in the tails of zebrafish embryos was marked within the box in (H). Scale bars: 200 µm. Data are presented as mean ± s.e.m. (n = 10). **P < 0.01, ***P < 0.001.

The interaction of platelets and tumor cells triggers tumor growth, migration and metastasis 38. To determine the effects of DOX-MPs/ASA@NF-induced inhibition of platelet activation on the proliferation of tumor cells, the platelets treated with PBS, NF, DOX-MPs, ASA, DOX-MPs@NF, ASA@NF or DOX-MPs/ASA@NF were co-incubated with 4T1 or H22 cells for 48 h, and the cell viability of 4T1 (Figure 5A) and H22 cells (Figure S11) was determined by CCK-8 assay. As expected, the platelets markedly increased the proliferation of 4T1 and H22 cells. NF, DOX-MPs or DOX-MPs@NF did not remarkably affect the platelet-promoted proliferation of tumor cells. However, the cell viability of 4T1 and H22 cells co-incubated with ASA, ASA@NF or DOX-MPs/ASA@NF-treated platelets was significantly lower compared with PBS-treated platelet group, suggesting that DOX-MPs/ASA@NF efficiently suppressed platelet-promoted tumor cell growth due to the platelet activation inhibition. The tumor cell apoptosis inhibition in serum-free medium by DOX-MPs/ASA@NF-suppressed platelet activation was further confirmed (Figure S12).

The activated platelets induce EMT of tumor cells, thus promoting tumor migration and metastasis 13. To investigate whether DOX-MPs/ASA@NF decreased platelet-triggered EMT, the expression of several relevant protein markers was determined in 4T1 cells after co-incubation with PBS-, NF-, DOX-MPs-, ASA-, DOX-MPs@NF-, ASA@NF- or DOX-MPs/ASA@NF-treated platelets by flow cytometry or western blot. As expected, the platelets significantly decreased E-cadherin expression (Figure 5B-C and Figure S13) and increased N-cadherin and Snail expression (Figure 5C and Figure S13), which is the main feature of EMT 39, confirming that the platelets facilitated EMT of 4T1 cells. However, the platelet-promoted decrease in E-cadherin expression (Figure 5B-C and Figure S13) and increase in N-cadherin and Snail expression (Figure 5C and Figure S13) in 4T1 cells were obviously abrogated after ASA-, ASA@NF- or DOX-MPs/ASA@NF treatment, implying that platelet-induced EMT in 4T1 cells could be weakened by DOX-MPs/ASA@NF.

To investigate the inhibitory effects of DOX-MPs/ASA@NF on the platelet-induced migration of tumor cells, a scratch wound healing assay was first performed in 4T1 cells (Figure 5D-E). 4T1 cells grown in the scratch test mold were co-incubated with PBS-, NF-, DOX-MPs-, ASA-, DOX-MPs@NF-, ASA@NF- or DOX-MPs/ASA@NF-treated platelets. Consistently, the migration of 4T1 cells was remarkably promoted by the platelets as evidenced by the almost migratory cell-covered wound in the PBS-treated group after 18 h. Treatment with NF-, DOX-MPs- or DOX-MPs@NF did not obviously affect platelet-promoted migration of 4T1 cells. However, the scratch still remained an obvious gap in ASA-, ASA@NF- or DOX-MPs/ASA@NF-treated group after 18 h, suggesting that DOX-MPs/ASA@NF efficiently suppressed platelet-promoted migration of tumor cells. Here, DOX-MPs/ASA@NF-treated platelets did not significantly affect the growth of 4T1 cells after co-culture for 18 h (Figure S14), excluding the possibility that the decreased migration of 4T1 cells by DOX-MPs/ASA@NF-inhibited platelet activation was due to the reduced tumor cell growth. The inhibition of platelet-promoted tumor cell migration by DOX-MPs/ASA@NF was further confirmed by the transwell migration assay (Figure 5F-G).

The effects of DOX-MPs/ASA@NF-inhibited platelet activation on the metastasis of 4T1 cells were evaluated in Is5Tg/+(AB) transgenic zebrafish (Figure 5H-I), which expressed mCherry in blood vessels. The platelets treated with PBS-, NF-, DOX-MPs-, ASA-, DOX-MPs@NF-, ASA@NF- or DOX-MPs/ASA@NF were co-injected with DIO-labeled 4T1 cells into the yolk sacs of the two days post-fertilization (2 dpf) zebrafish embryos. At 72 h after injection, the zebrafish tails were observed by confocal microscope since tumor cells prefer to metastasize to the caudal hematopoietic tissues of zebrafish 40. Expectedly, co-injection of the platelets significantly increased the colonization of 4T1 cells in the zebrafish tail, as indicated by the stronger DIO fluorescence in the co-injection group of PBS-treated platelets and 4T1 cells compared with 4T1 cell injection group, revealing that the platelets facilitated the metastasis capacity of 4T1 cells. NF-, DOX-MPs- or DOX-MPs@NF-treated platelets exhibited the similar effects of promoting metastasis of 4T1 cells. However, significant lower metastatic 4T1 cells were found in the zebrafish tails after co-injection with ASA-, ASA@NF or DOX-MPs/ASA@NF-treated platelets, indicating that DOX-MPs/ASA@NF-inhibited platelet activation significantly suppressed platelet-promoted tumor metastasis.

### DOX-MPs/ASA@NF inhibits postsurgical tumor recurrence and metastasis in orthotopic breast tumor model

When the tumor volume of orthotopic 4T1 tumor-bearing mice, a breast tumor model with spontaneous metastases, reached about 300 mm^3^, the tumors were surgically resected up to 90%. DOX, DOX-MPs, DOX-MPs@NF or DOX-MPs/ASA@NF was then implanted into the tumor resection cavity and the *in vivo* DOX biodistribution was determined (Figure S15). More DOX was detected in DOX-MPs@NF- and DOX-MPs/ASA@NF-treated groups compared with free DOX- and DOX-MPs-treated groups, suggesting that loading DOX-MPs to NF could increase DOX retention in tumor resection cavity.

**Figure 6:**
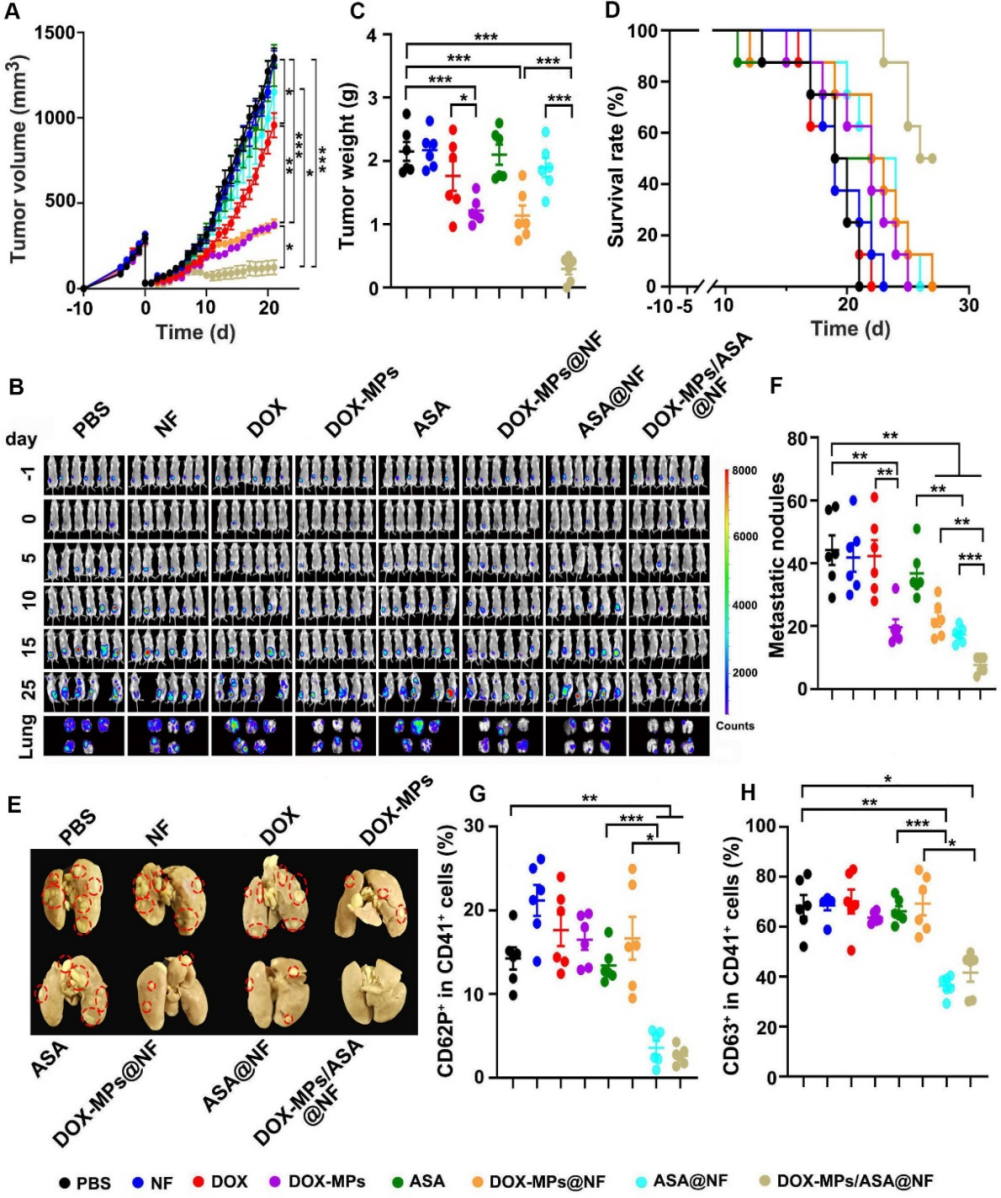
** Anti-recurrence/metastasis activity of DOX-MPs/ASA@NF in 4T1-Luc tumor-bearing mice after surgical tumor resection. (A)** Average tumor growth curves of 4T1-Luc tumor-bearing mice undergoing surgical tumor resection after implanting PBS, NF, DOX, DOX-MPs, ASA, DOX-MPs@NF, ASA@NF or DOX-MPs/ASA@NF at the DOX dosage of 1 mg kg^-1^ and ASA dosage of 75 mg kg^-1^ into the tumor resection cavity. **(B)** *In vivo* bioluminescence imaging of 4T1-Luc tumor-bearing mice undergoing surgical tumor resection at the different time points after treatment as above. The lungs were harvested at 25 days after treatment. **(C)** Tumor weight of 4T1-Luc tumor-bearing mice undergoing surgical tumor resection at 25 days after treatment as above. Data are presented as mean ± s.e.m. (n = 6). **(D)** The survival time of 4T1-Luc tumor-bearing mice undergoing surgical tumor resection after treatment as above (n=8). **(E,F)** Lung images (E) and metastatic nodule numbers in lungs (F) of 4T1-Luc tumor-bearing mice at 25 days after treatment as above. Red circles in (E) marked the typic tumor nodules. **(G,H)** Percentage of CD62P^+^ (G) and CD63^+^ cells (H) in CD41^+^ platelets of tumor tissues of 4T1-Luc tumor-bearing mice undergoing surgical tumor resection at 25 days after treatment as above. Data are presented as mean ± s.e.m. (n = 6). *P < 0.05, **P < 0.01, ***P < 0.001.

Combined with the efficient platelet deactivation capacity, the postsurgical anti-recurrence/metastasis activity of DOX-MPs/ASA@NF was evaluated in orthotopic breast tumor model inoculating luciferase-expressing 4T1 (4T1-Luc) cells after implanting PBS, NF, DOX, DOX-MPs, ASA, DOX-MPs@NF, ASA@NF or DOX-MPs/ASA@NF into the tumor resection cavity. The primary tumors of mice treated with PBS, NF, ASA or ASA@NF grew fast and exhibited the similar tumor growth curves (Figure 6A). However, DOX-MPs/ASA@NF exhibited the strongest anti-recurrence capacity after surgical tumor resection, with 1 of the 6 mice becoming tumor free (Figure 6A and Figure S16). Moreover, the negligible bioluminescence signals (Figure 6B) and the minimum tumor weight (Figure 6C) were detected in DOX-MPs/ASA@NF-treated group. DOX-MPs/ASA@NF-treated mice exhibited the longest survival time, with 50% of mice alive at 27 days after treatment although the mice in other groups had died (Figure 6D). Histological analysis by H&E staining demonstrated severe tumor necrosis in DOX-MPs/ASA@NF-treated mice (Figure S17A). Moreover, the minimum proportion of Ki67-positive proliferating tumor cells and the maximum proportion of TUNEL-positive apoptotic tumor cells was detected in the DOX-MPs/ASA@NF-treated mice (Figure S17B-C), further confirming the excellent postsurgical anti-recurrence capacity of DOX-MPs/ASA@NF. In addition, *ex vivo* lung tissue images showed that DOX-MPs/ASA@NF-treated group exhibited the lowest bioluminescence intensity (Figure 6B). Meanwhile, the significantly fewer metastatic nodules in the lungs were confirmed in DOX-MPs/ASA@NF-treated group by both counting nodule numbers (Figure 6E-F) and H&E staining (Figure S18), suggesting that DOX-MPs/ASA@NF efficiently inhibited lung metastasis of 4T1-Luc tumor-bearing mice after surgical resection. In addition, DOX-MPs/ASA@NF efficiently induced immunogenic cell death (ICD), as evidenced by the enhanced expression of calreticulin (CRT) and high mobility group box 1 (HMGB1) (Figure S19), the typical ICD markers in tumor tissues, suggesting that DOX-MPs/ASA@NF might induce antitumor immunity. As anticipated, immunofluorescence analysis showed that DOX-MPs/ASA@NF treatment significantly decreased the expression of platelet marker CD41 and activated platelet marker CD62P in tumor tissues (Figure S20). Concurrently, the ratio of CD62P^+^CD41^+^ (Figure 6G) and CD63^+^CD41^+^ cells (Figure 6H) was substantially inhibited in tumor tissues of DOX-MPs/ASA@NF-treated group. These results indicated that DOX-MPs/ASA@NF prevented postsurgical tumor recurrence and metastasis possibly by killing tumor cells and inhibiting platelet activation. No significant toxicity was detected in DOX-MPs/ASA@NF-treated group, as indicated by body weight (Figure S21), H&E staining of major organs (Figure S22) and serological analysis (Figure S23). Meanwhile, DOX-MPs/ASA@NF did not affect the number of hemocytes, including platelets of these treated mice (Figure S24). These results revealed that DOX-MPs/ASA@NF had good biocompatibility and biosafety.

### DOX-MPs/ASA@NF inhibits postsurgical tumor recurrence in subcutaneous liver tumor model

**Figure 7:**
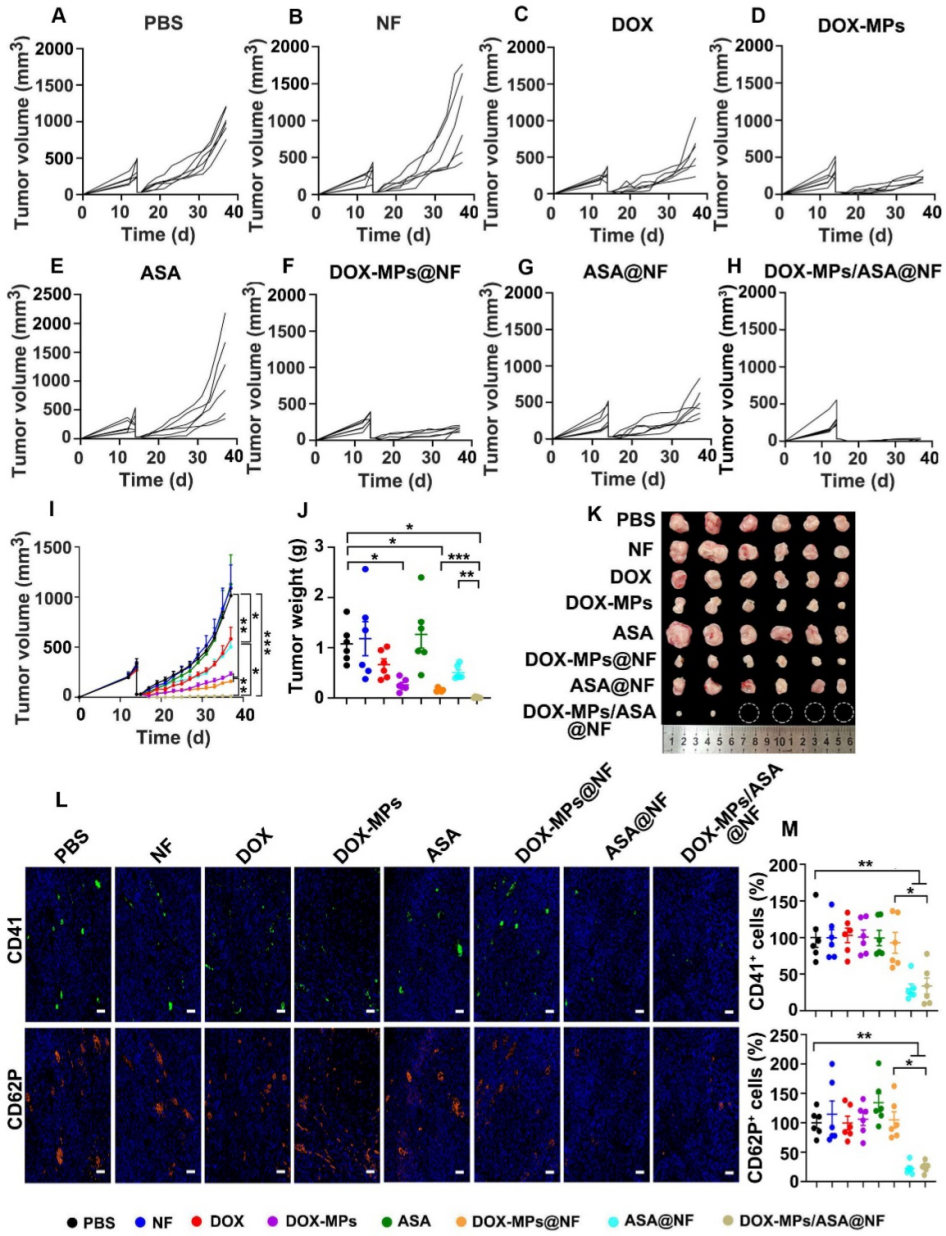
** Anti-recurrence activity of DOX-MPs/ASA@NF in H22 tumor-bearing mice after surgical tumor resection. (A-H)** Individual tumor growth curves of H22 tumor-bearing mice undergoing surgical tumor resection after implanting PBS (A), NF (B), DOX (C), DOX-MPs (D), ASA (E), DOX-MPs@NF (F), ASA@NF (G) or DOX-MPs/ASA@NF (H) at the DOX dosage of 1 mg kg^-1^ and ASA dosage of 75 mg kg^-1^ into the tumor resection cavity. **(I)** Average tumor growth curves of H22 tumor-bearing mice undergoing surgical tumor resection after treatment as above. **(J,K)** Tumor weight (J) and images (K) of H22 tumor-bearing mice undergoing surgical tumor resection at 24 days after treatment as above. Data are presented as mean ± s.e.m. (n = 6). *P < 0.05, **P < 0.01, ***P < 0.001. **(L,M)** Representative immunofluorescent staining images of CD41 and CD62P (L) and the quantification of CD41^+^ and CD62P^+^ cells (M) in tumor tissues of H22 tumor-bearing mice undergoing surgical tumor resection at 24 days after treatment as above. Scale bars: 50 µm. Data are presented as mean ± s.e.m. (n = 6). *P < 0.05, **P < 0.01.

The effects of DOX-MPs/ASA@NF on preventing postoperative tumor recurrence were further evaluated in subcutaneous liver cancer models bearing H22 tumors (Figure 7A-I). Consistently, treatment with DOX-MPs and DOX-MPs@NF significantly halted the development and progression of the tumor mass, with 77.3% and 84.3% tumor inhibition, respectively. However, DOX-MPs/ASA@NF exhibited the strongest inhibitory effect, with 4 out of 6 mice having no tumor recurrence. The average weight of the tumor tissues excised at the end of treatment also exhibited the same trend (Figure 7J-K). The worst tumor necrosis (Figure S25A), the lowest proliferation and highest apoptosis of tumor cells (Figure S25B-C) were detected in DOX-MPs/ASA@NF-treated group, further confirming the excellent postsurgical anti-recurrence capacity of DOX-MPs/ASA@NF. Immunofluorescence staining analysis showed that the expression of CD41 and CD62P in tumors was significantly decreased in DOX-MPs/ASA@NF-treated group (Figure 7L-M), suggesting that the decreased platelet activation might be responsible for the enhanced tumor inhibition in DOX-MPs/ASA@NF-treated group. Body weight change (Figure S26), H&E staining of major organs (Figure S27), serological (Figure S28) and blood cell analysis (Figure S29) in H22 tumor-bearing mice further confirmed the good biosafety of DOX-MPs/ASA@NF.

## Conclusions

In summary, the biocompatible PLGA electrospun NF films loading DOX-MPs and ASA are constructed to prevent postoperative tumor recurrence and metastasis. The PLGA NF films significantly enhance the tumor accumulation of DOX-MPs, facilitating killing the residual tumor cells. In addition, the sustainedly released ASA efficiently reduces the interaction between platelets and tumor cells and inhibits the platelet activation, decreasing the platelet-promoted proliferation, migration and metastasis of tumor cells. As a result, DOX-MPs/ASA@NF remarkably controls the local tumor recurrence and distal metastasis following surgical resection. DOX-MPs/ASA@NF may be a potential candidate for postoperative in-situ treatment.

## Methods

### Materials

Fetal bovine serum (FBS) and collagenase Type I were obtained from Gibco Life Technologies (New York, NY, USA). Roswell Park Memorial Institute (RPMI) 1640 medium, PBS and penicillin-streptomycin were purchased from HyClone (Logan, Utah, USA). PLGA (75/25, Mw = 159000) was purchased from Jinan Daigang Biomaterial Co., Ltd (Jinan, China). ASA and AA were purchased from Aladdin (Shanghai, China). Doxorubicin hydrochloride was purchased from Meilunbio (Dalian, China). Antibodies used for flow cytometric analysis were purchased from BioLegend (San Diego, CA, USA). All other reagents were of analytical grade and used without any further purification.

### Cell culture and animals

The murine hepatocarcinoma cell line H22 and murine breast cancer cell line 4T1 were purchased from Type Culture Collection of the Chinese Academy of Sciences (Shanghai, China). 4T1-Luc cell line was kindly provided by Dr. Yinsong Wang (Tianjin Medical University, Tianjin, China). The cells were cultured in RPMI 1640 medium supplemented with 10% FBS and 1% penicillin-streptomycin at 37 °C under 5% CO_2_. TRCs were selected in soft 3D fibrin gels (90 Pa) as described 24. Briefly, 1×10^4^ parental tumor cells (including H22 and 4T1 cells) and fibrinogen at the final concentration of 1 mg mL^-1^ were mixed well, and then added into the wells of a 24-well plate preadded with 5 µL thrombin (0.1 U µL^-1^). The plate was incubated in a cell culture incubator for 30 min and then 1 mL RPMI 1640 medium containing 10% FBS was added. On day 5, the spheroids in soft 3D fibrin gels were harvested, digested with 0.4% (w/v) dispase II for 10 min at 37 °C to single cells and then cultured in serum-free DMEM/F-12 medium supplemented with 2% B27, 20 ng mL^-1^ mouse epidermal growth factor (mEGF) and 1% glutamine.

BALB/c mice (male and female, 5-6 weeks of age) were purchased from Beijing Vital River Laboratory Animal Technology Co., Ltd. (Beijing, China). H22 tumor-bearing mice were constructed by subcutaneous injection of 2×10^6^ H22 cells into the flanks of male BALB/c mice. 4T1 tumor-bearing mice were constructed by orthotopic inoculation of 5×10^5^ 4T1-Luc cells into the right forth breast fat pad of BALB/c female mice. All animal experiments were performed under the guidance approved by the Institutional Animal Care and Use Committee at Tongji Medical College, Huazhong University of Science and Technology (Wuhan, China).

### Preparation of DOX-MPs

To collect DOX-MPs 26, TRCs (including H22 and 4T1 TRCs) were treated with 200 µg mL^-1^ DOX and then irradiated with ultraviolet irradiation (UVB, 300 J m^-2^) for 1 h. At 12 h after treatment, the supernatants were centrifuged at 1000*g* for 10 min and 14,000*g* for 2 min to remove cells and cell debris, followed by centrifugating at 14,000*g* for 60 min to collect DOX-MPs. The pellets were washed with PBS and resuspended in cell culture medium for the following experiments. DOX content in MPs was measured by high performance liquid chromatography (HPLC). The hydrodynamic diameter and zeta potential of DOX-MPs were measured by DLS on a Zetasizer Nano ZS90 instrument (Malvern Instruments, UK). The morphology of DOX-MPs was observed by a HT 7700 TEM (Hitachi, Tokyo, Japan).

### Preparation of DOX-MPs/ASA@NF

A single-axial electrospinning technique was used to prepare PLGA NF films and ASA@NF. The electrospinning solution containing 18% (w/v) PLGA without or with 1.8% (w/v) ASA in chloroform was added into a plastic syringe with a home-made single spinneret. A syringe pump (KDS100, Cole-Parmer, USA) was used to drive the working fluids at a constant rate of 1 mL h^-1^. A roller (30 rpm min^-1^) covered with aluminum foil was grounded and used as the collector. A high-voltage power supply (TESLAMAN, China) was employed to provide a potential difference between the spinneret and the collector. After optimization, the applied voltage and spinneret-to-collector distance were fixed at 18 kV and 20 cm, respectively. After electrospinning, NF and ASA@NF were collected and dried in a constant temperature vacuum oven for 24 h. NF and ASA@NF were cut into round patches with a diameter of 13 mm and then put into a filter as a filter membrane. DOX-MPs@NF and DOX-MPs/ASA@NF were acquired by pushing DOX@MPs through the filter using an injector.

### Characterization of DOX-MPs/ASA@NF

The morphology of DOX-MPs/ASA@NF was determined by a HT 7700 TEM and a Sirion 200 SEM (FEI, The Netherlands). The diameter of DOX-MPs/ASA@NF was expressed as the mean values of random samples (n = 100) from the SEM images via image J software. The porosity of DOX-MPs/ASA@NF was estimated by liquid intrusion method 41. Briefly, the dry DOX-MPs/ASA@NF which had been weighed was soaked in ethanol for 2 h and reweighed. The porosity was calculated according to the equation: porosity (%) = V_ethanol_/(V_ethanol_ + V_DOX-MPs/ASA@NF_). The *in vitro* drug release of DOX-MPs/ASA@NF was determined by putting DOX-MPs/ASA@NF at the DOX concentration of 4 μg mL^-1^ and ASA concentration of 300 μg mL^-1^ in PBS at the shaking speed of 100 rpm on a constant temperature shaker (37 °C), and the release amount of DOX and ADS was then quantified by HPLC at the predetermined intervals.

The *in vitro* degradation of NF was determined by placing 3 mg NF in 10 mL of PBS containing 8 μg mL^-1^ Proteinase K and 4 mg mL^-1^ lysozyme (pH 7.4) at the shaking speed of 100 rpm on a constant temperature shaker (37 °C). At the predetermined intervals, NF were taken out, washed three times with PBS, lyophilized and weighed. The *in vivo* degradation of NF was determined by surgically implanting NF into the flanks of BALB/c mice, and the residual NF were collected and photographed at the predetermined time points.

The *in vitro* coagulation of NF was determined by placing the same volume of NF or the commercially available gelatin sponge in the culture dish, followed by adding 100 μL of fresh sodium citrate-anticoagulated mouse blood when mixing with 10 µL CaCl_2_ (0.2 mol L^-1^) and then incubating at 37 °C for 10 min. 50 mL of distilled water was carefully added into the culture dish along the edge, and the absorbance at 545 nm was measured on a Multiskan FC microplate reader (Thermo Scientific, Waltham, MA, USA).

### Cell viability and apoptosis assay

4T1 or H22 cells were treated with PBS, NF, DOX, DOX-MPs, ASA, DOX-MPs@NF or DOX-MPs/ASA@NF at the DOX concentration of 2 μg mL^-1^ and ASA concentration of 150 μg mL^-1^. At 24 h after treatment, the cells were washed twice with PBS. The cell viability was determined using CCK-8 assay (Dojindo, Kumamoto, Kyushu, Japan). The apoptosis was measured by Annexin V apoptosis kit with 7-AAD and Calcein-AM/PI double stain kit (Yeasen, Shanghai, China) according to the manufacturer's instructions.

### Platelet isolation

The platelet-rich plasma (PRP) was obtained by centrifuging fresh blood from the mice eye socket at 180*g* for 15 min at 25 °C. The PRP was further centrifuged at 1300*g* for 15 min at 25 °C and then the pellets were collected to obtain the platelets. The platelets were washed twice with PBS containing 0.25 µΜ prostaglandin E1 (PGE1) and then counted using a blood cell counting board.

### DOX-MPs/ASA@NF-induced inhibition of platelet aggregation

PRP was treated with PBS, NF, DOX-MPs, ASA, DOX-MPs@NF, ASA@NF or DOX-MPs/ASA@NF at the DOX concentration of 8 μg mL^-1^ and ASA concentration of 600 μg mL^-1^ for 12 h. Then the treated PRP was incubated with 0.5 mM AA for 15 min. The platelet aggregation was reflected by the change of ultraviolet absorbance at 650 nm before and after AA addition using a Multiskan FC microplate reader.

The platelets were treated with PBS, NF, DOX-MPs, ASA, DOX-MPs@NF, ASA@NF or DOX-MPs/ASA@NF at the DOX concentration of 8 μg mL^-1^ and ASA concentration of 600 μg mL^-1^ for 12 h. The platelets were washed with PBS and then labeled with anti-CD41-APC or anti-CD61-PE for 30 min, respectively. The above anti-CD41-APC-labeled platelets (50 µL) were then mixed with anti-CD61-PE labeled platelets (50 µL) in the presence of 0.5 mM AA at 37 °C for 15 min. The cells were fixed with 4% paraformaldehyde and the ratio of CD41^+^CD61^+^ platelets in the mixtures was analyzed by flow cytometry (FC500, Beckman Coulter, Fullerton, CA, USA) 42.

### DOX-MPs/ASA@NF-induced inhibition of platelet activation

The platelets were treated with PBS, NF, DOX-MPs, ASA, DOX-MPs@NF, ASA@NF or DOX-MPs/ASA@NF at the DOX concentration of 8 μg mL^-1^ and ASA concentration of 600 μg mL^-1^ for 12 h. The above treated platelets were stimulated with 0.5 mM AA or 4T1 cells at a ratio of 1000 for 15 min. The platelets were stained with anti-CD41-FITC and anti-CD62P-PC7 or anti-CD63-APC and then detected by flow cytometry.

### DOX-MPs/ASA@NF-induced inhibition of platelet adhesion with tumor cells

The platelets were treated with PBS, NF, DOX-MPs, ASA, DOX-MPs@NF, ASA@NF or DOX-MPs/ASA@NF at the DOX concentration of 8 μg mL^-1^ and ASA concentration of 600 μg mL^-1^ for 12 h. The above treated platelets were labeled with anti-CD41-APC and then co-incubated with CFSE-labeled 4T1 cells at a ratio of 1000:1 for 8 h. The cells were fixed with 4% paraformaldehyde, washed with PBS and the ratio of CFSE^+^CD41^+^ cells was then detected by flow cytometry. For confocal microscopic analysis, the above treated platelets were labeled with DID and then co-incubated with DIO-labeled 4T1 cells at a ratio of 1000:1 for 8 h. The cells were fixed with 4% paraformaldehyde, washed with PBS and then observed by Olympus FV3000 confocal microscope (Tokyo, Japan).

### Effects of DOX-MPs/ASA@NF-induced inhibition of platelet activation on the proliferation and apoptosis of tumor cells

The platelets were treated with PBS, NF, DOX-MPs, ASA, DOX-MPs@NF, ASA@NF or DOX-MPs/ASA@NF at the DOX concentration of 8 μg mL^-1^ and ASA concentration of 600 μg mL^-1^ for 12 h. The above treated platelets were washed with PBS and then co-incubated with 4T1 or H22 cells at a ratio of 1000:1 in the serum-free medium for different time intervals. The cells were washed with PBS to remove the platelets. The cell viability was detected using CCK-8 assay according to the manufacturer's instructions. The apoptosis of cells was detected using Annexin V-FITC/PI apoptosis detection kit.

### Effects of DOX-MPs/ASA@NF-induced inhibition of platelet activation on the expression of EMT markers

The platelets were treated with PBS, NF, DOX-MPs, ASA, DOX-MPs@NF, ASA@NF or DOX-MPs/ASA@NF at the DOX concentration of 8 μg mL^-1^ and ASA concentration of 600 μg mL^-1^ for 12 h. 4T1 cells were co-incubated with the pretreated platelets for 24 h and then washed with PBS to remove the platelets. The expression of E-cadherin was detected by flow cytometry using anti-CD324-BV421 antibody and western blot, and the expression of N-cadherin and Snail was detected by western blot. For western blot analysis, 4T1 cells were lysed in lysis buffer and the total protein concentration was measured by a BCA kit (Beyotime Biotechnology, Shanghai, China) according to the manufacturer's instruction. 20 μg of lysates were separated by sodium dodecyl sulfate-polyacrylamide gel electrophoresis (SDS-PAGE) and transferred onto PVDF membranes. The membranes were blocked with 5% fat-free milk for 2 h, and then incubated with anti-E-cadherin, anti-N-cadherin and anti-Snail (Cell Signaling Technology, Danvers, MA) at 4 °C overnight. After washing with Tris-buffered saline containing 0.1% Tween-20 (TBST), the membranes were incubated with anti-rabbit IgG (H+L) (DyLight^TM^ 800 4 × PEG Conjugate) or anti-mouse IgG (H+L) (DyLight^TM^ 800 Conjugate) at 37 °C for 2 h. The protein bands were detected using Odyssey CLx imager (Li-COR Biosciences, Lincoln, NE, USA).

### Effects of DOX-MPs/ASA@NF-induced inhibition of platelet activation on the migration of tumor cells

The platelets were treated with PBS, NF, DOX-MPs, ASA, DOX-MPs@NF, ASA@NF or DOX-MPs/ASA@NF at the DOX concentration of 8 μg mL^-1^ and ASA concentration of 600 μg mL^-1^ for 12 h. 4T1 cells were seeded in the scratch test mold and incubated overnight. The above treated platelets were added to the well and then co-incubated with 4T1 cells at a ratio of 1000:1 in the serum-free medium. At the predetermined intervals, the scratch was observed and photographed under an inverted microscope. The scratch area was counted using image J software.

The migration capability of 4T1 cells was further evaluated by the migration transwell assay. Briefly, the platelets were treated with PBS, NF, DOX-MPs, ASA, DOX-MPs@NF, ASA@NF or DOX-MPs/ASA@NF at the DOX concentration of 8 μg mL^-1^ and ASA concentration of 600 μg mL^-1^ for 12 h. 4T1 cells were seeded on the upper chambers in the serum-free medium and RPMI 1640 medium containing 10% FBS was added into the lower chambers. The above treated platelets were added to the upper chambers and co-incubated with 4T1 cells at a ratio of 1000:1 for 12 h. The transwell chambers were removed, and the invaded 4T1 cells were fixed with 4% paraformaldehyde, stained with 0.1% crystal violet and counted under an inverted microscope.

### Effects of DOX-MPs/ASA@NF-induced inhibition of platelet activation on the metastasis of tumor cells in zebrafish embryos

Is5Tg/+(AB) transgenic zebrafish were kindly provided by Dr. Mugen Liu (Huazhong University of Science and Technology, Wuhan, China). The platelets were treated with PBS, NF, DOX-MPs, ASA, DOX-MPs@NF, ASA@NF or DOX-MPs/ASA@NF at the DOX concentration of 8 μg mL^-1^ and ASA concentration of 600 μg mL^-1^ for 12 h. The above treated platelets were co-injected with DIO-labeled 4T1 cells at a ratio of 1000:1 into the yolk sacs of zebrafish embryos (250 4T1 cells embryo^-1^) at 48 h post-fertilization. After injection, the embryos were maintained at 28 °C for 1 h and then incubated at 35 °C for 72 h. The migratory cells in the tail of the zebrafish were observed and photographed by Olympus FV3000 confocal microscope. The fluorescence of 4T1 cells was quantified using image J software.

### Anti-recurrence and metastasis of DOX-MPs/ASA@NF in 4T1 and H22 tumor-bearing mice after surgical tumor resection

When tumor volume of 4T1-Luc or H22 tumor-bearing mice reached around 300 mm^3^, the mice were randomly divided into eight groups and then the tumors were removed to only 10% left. After surgery, PBS, NF, DOX, DOX-MPs, ASA, DOX-MPs@NF, ASA@NF or DOX-MPs/ASA@NF at the DOX dosage of 1 mg kg^-1^ and ASA dosage of 75 mg kg^-1^ was placed in the tumor resection cavity. The tumor volume was measured with a vernier caliper every day and the mice of 4T1-Luc tumor-bearing mice were also observed using an *in vivo* bioluminescence imaging system every five days. At the indicated time intervals, the mice were euthanized and the organs (heart, liver, spleen, lung, and kidney) and tumors were harvested. The tumors were weighted, fixed with 4% paraformaldehyde, sectioned, and stained with H&E, Ki67 and TUNEL for anticancer treatment evaluation and anti-CD41 or anti-CD62P antibody for platelet analysis. The lungs of 4T1-Luc tumor-bearing mice were fixed in Bouin's solution (Solarbio, Beijing, China) for 12 h and the metastatic tumor nodules were counted. The organs were fixed with 4% paraformaldehyde, sectioned and stained with H&E. The expression of CRT and HMGB1 in the tumors was performed by immunohistochemistry. To further analyze the platelets in the tumors, the tumors were cut into pieces, incubated with RPMI 1640 media containing 0.8 mg mL^-1^ collagenase and 5 μg mL^-1^ DNase I for 60 min at 37 °C and then passed through a 40 μm nylon mesh. The single cells were labeled with anti-CD41-FITC, anti-CD62P-PC7 and anti-CD63-APC for 30 min according to the instructions and then detected by flow cytometry.

### Statistical analysis

All data were expressed as the mean ± standard error of the mean (s.e.m) of at least three samples. Statistical analyses were performed using GraphPad Prism software and SPSS software. Comparison between multiple groups was used by One-way analysis of variance (ANOVA). *P* < 0.05 was considered statistically significant.

## Supplementary Material

Supplementary figures.
