# Supplementary material for: Biodegradable electrospun nanofibrous platform integrating antiplatelet therapy-chemotherapy for preventing postoperative tumor recurrence and metastasis
Source: Theranostics. 2022 Apr 24;12(7):3503–17. doi: 10.7150/thno.69795 (PMC9065196; doi:10.7150/thno.69795)
Supplement: Supplementary file 1 — Supplementary figures. [file thnov12p3503s1.pdf]

# Biodegradable electrospun nanofibrous platform integrating antiplatelet therapy-chemotherapy for preventing postoperative tumor recurrence and metastasis

Jianye Li[a,1], Jiaojiao Li[a,1], Yuzhu Yao[a,1], Tuying Yong[a], Nana Bie[a], Zhaohan Wei[a], Xin Li[a], Shiyu Li[a], Jiaqi Qin[a], Haibo Jia[b], Qing Du [a,b,c,d*], Xiangliang Yang[a,b,c,d*] and Lu Gan[a,b,c,d*]

[a]*National Engineering Research Center for Nanomedicine, College of Life Science and Technology, Huazhong University of Science and Technology, Wuhan 430074, China*

[b]*Key Laboratory of Molecular Biophysics of the Ministry of Education, College of Life Science and Technology, Huazhong University of Science and Technology, Wuhan 430074, China*

[c]*Hubei Key Laboratory of Bioinorganic Chemistry and Materia Medica, Huazhong University of Science and Technology, Wuhan 430074, China*

[d]*Hubei Engineering Research Center for Biomaterials and Medical Protective Materials, Huazhong University of Science and Technology, Wuhan 430074, China*

[1]These authors contributed equally to this work.

*Correspondence should be addressed to: lugan@mail.hust.edu.cn (L. Gan), yangxl@mail.hust.edu.cn (X. Yang), qing_du@mail. hust.edu.cn (Q. Du).

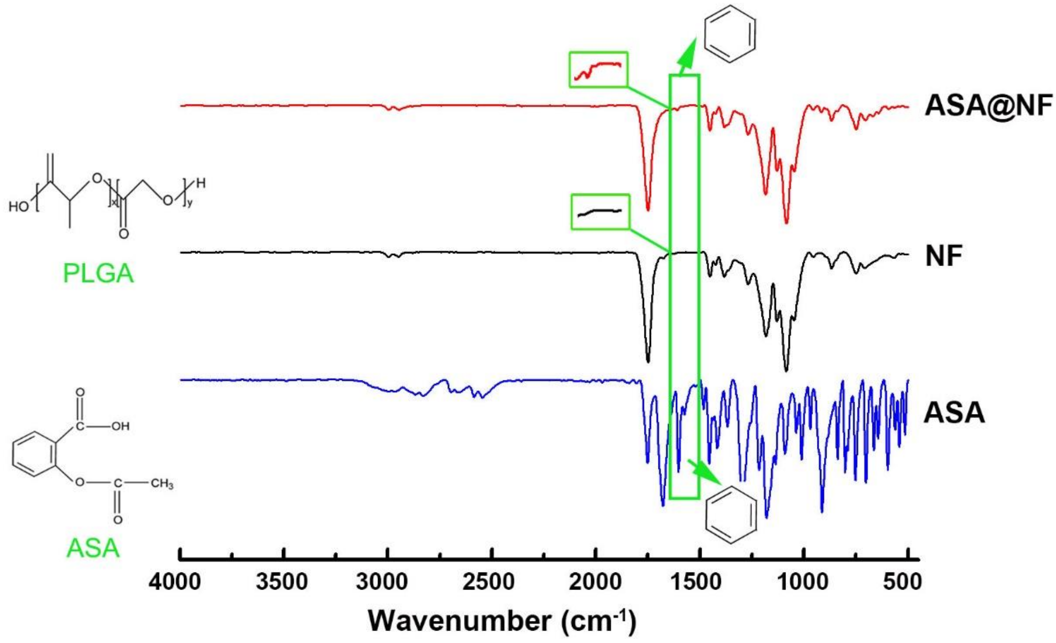


**Figure S1.** The FTIR of ASA, NF and ASA@NF. ASA and ASA@NF exhibited the peaks at 1602 $cm^{-1}$, which can be attributed to the C–C stretching vibration in the aromatic ring of ASA.

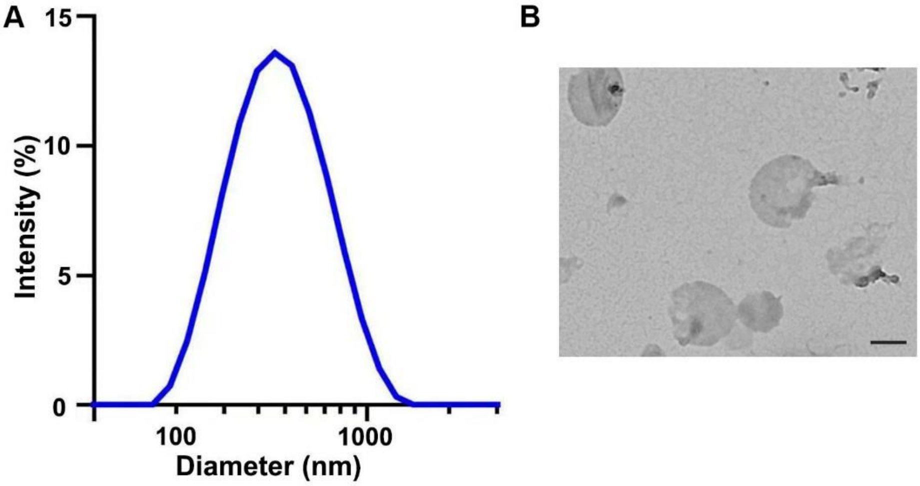


**Figure S2.** Characterization of DOX-MPs. (A) The diameter of DOX-MPs by DLS analysis. (B) The morphology of DOX-MPs by TEM. Scale bar: 200 nm.

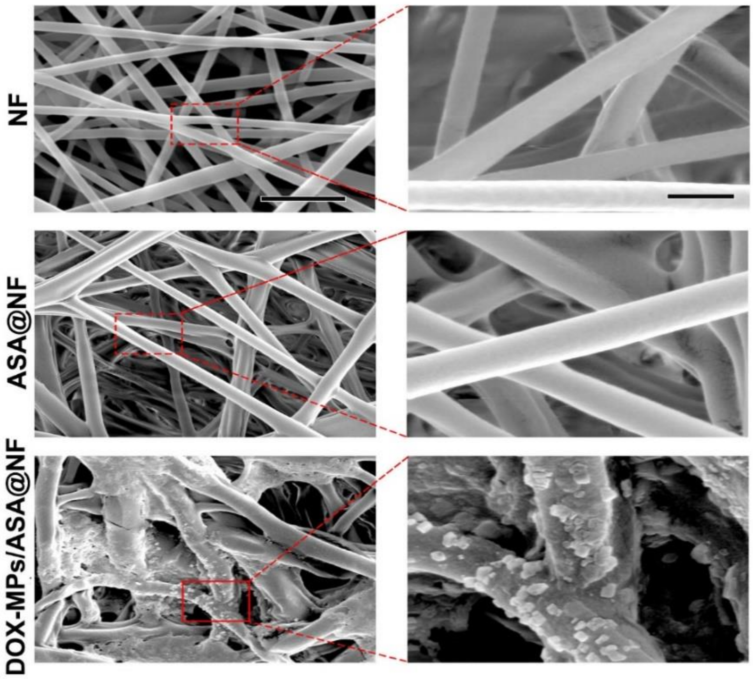


**Figure S3.** SEM images of NF, ASA@NF and DOX-MPs/ASA@NF. The right images were the amplification of the insets in the left images. Scale bars: 5 μm (left) and 1 μm (right).

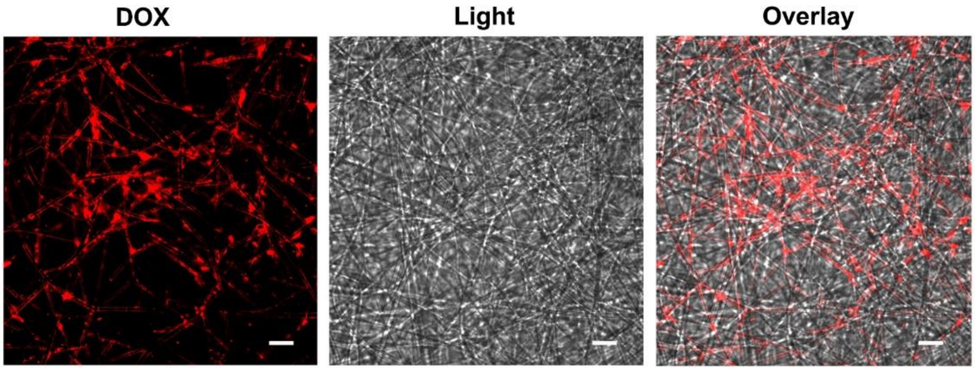


**Figure S4.** Colocalizaiton of DOX-MPs fluorescence with ASA@NF in DOX-MPs/ASA@NF. DOX-MPs fluorescence was detected by confocal microscopy and ASA@NF was observed under light microscopy. Scale bars: 10 μm.

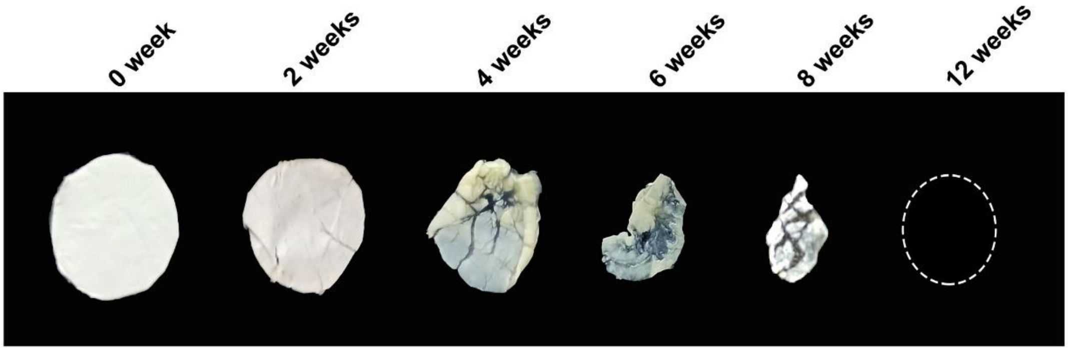


**Figure S5.** Images of PLGA NF films at different time points after subcutaneous implantation into the flanks of BALB/c mice.

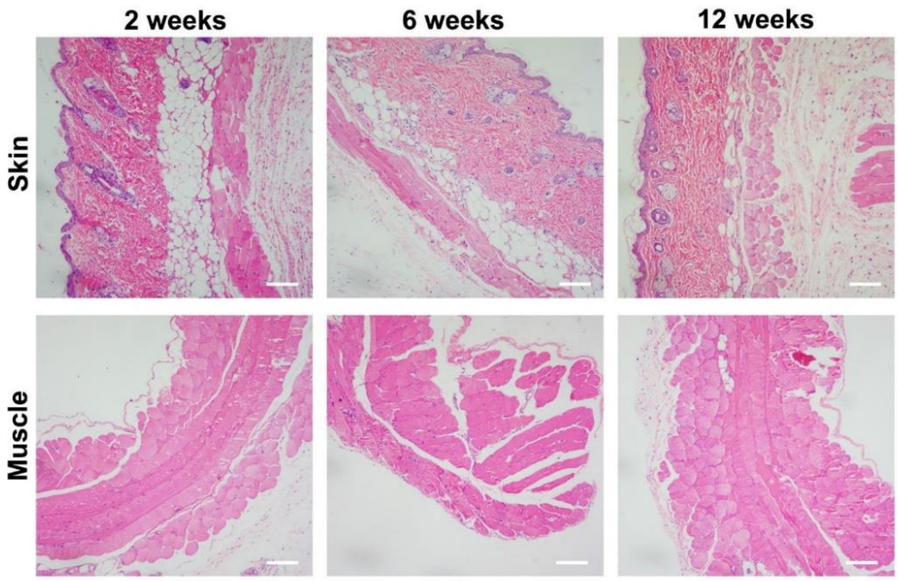


**Figure S6.** H&E staining of surrounding skins and abdominal muscle tissues at different time intervals after subcutaneous implantation of PLGA NF films into the flanks of BALB/c mice. Scale bars: 50 μm.

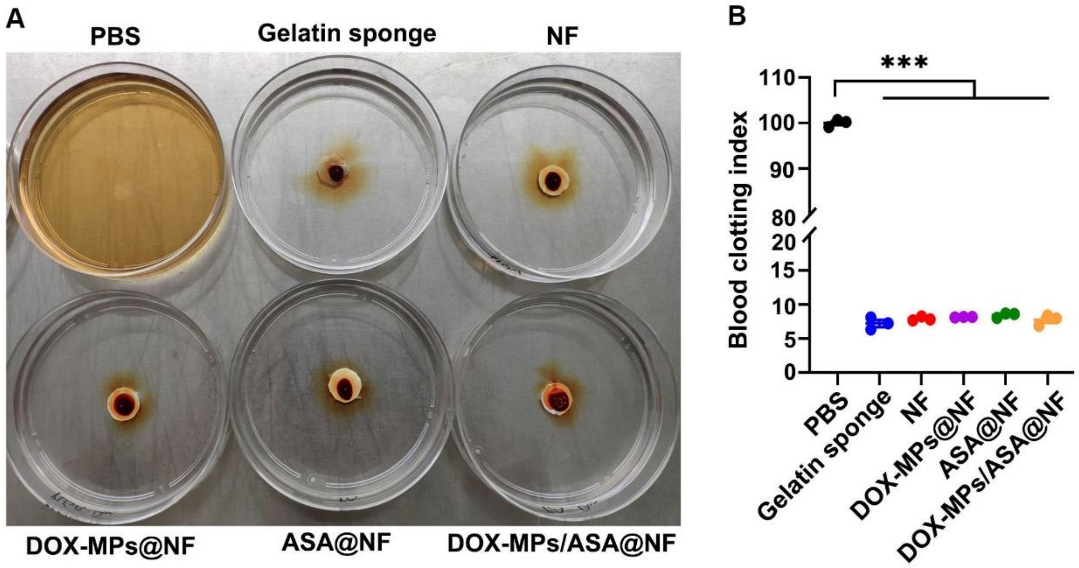


**Figure S7.** In vitro blood-clotting ability of DOX-MPs/ASA@NF. (A) Representative images of PBS, gelatin sponge, NF, DOX-MPs@NF, ASA@NF or DOX-MPs/ASA@NF after incubation with sodium citrate-anticoagulated mouse blood mixing with 0.2 mol $L^{-1}$ $CaCl_2$ at 37 °C for 10 min. (B) The blood clotting index of the above materials as measured by the absorbance at 545 nm after treatment as above. Data are presented as mean ± s.e.m. (n = 3). ***$P < 0.001$.

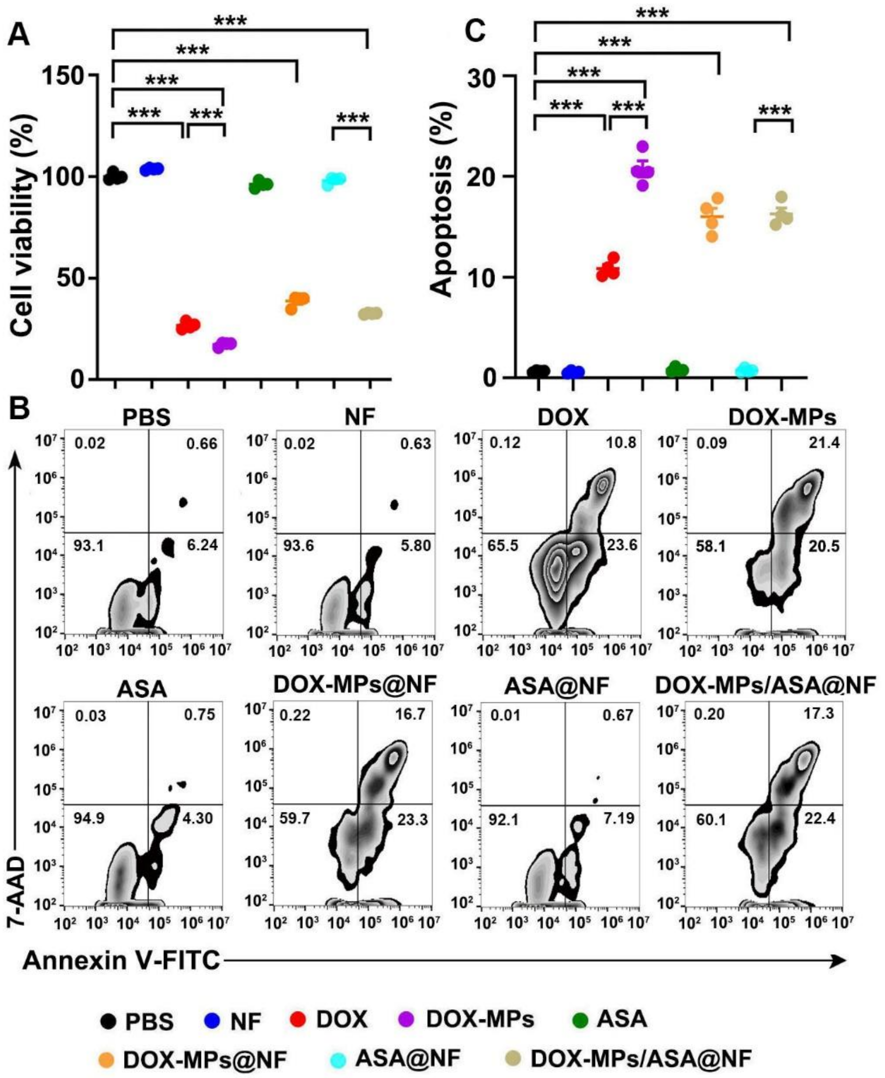


**Figure S8.** In vitro cytotoxicity of DOX-MPs/ASA@NF against H22 cells. (A) Cell viability of H22 cells after treatment with PBS, NF, DOX, DOX-MPs, ASA, DOX-MPs@NF, ASA@NF or DOX-MPs/ASA@NF at the DOX concentration of 2 μg $mL^{-1}$ and ASA concentration of 150 μg $mL^{-1}$ for 24 h by CCK-8 assay. (B,C) Representative flow cytometric images (B) and the quantification (C) of apoptosis in H22 cells after treatment as above by Annexin V apoptosis kit with 7-AAD. Data are presented as mean ± s.e.m. (n = 4). ***$P < 0.001$.

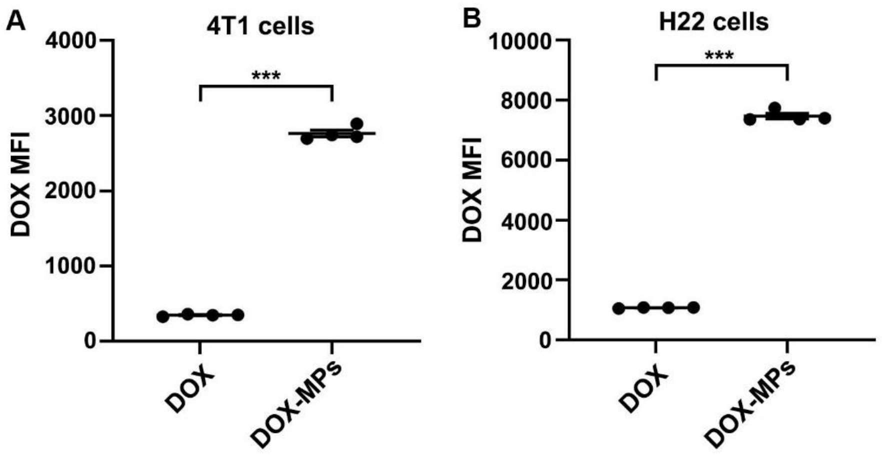


**Figure S9.** Cellular uptake of DOX-MPs by tumor cells. (A,B) DOX mean fluorescence intensity (MFI) in 4T1 (A) and H22 (B) cells after treatment with DOX or DOX-MPs at the DOX concentration of 2 μg $mL^{-1}$ for 4 h. Data are presented as mean ± s.e.m. (n = 4). ***$P < 0.001$.

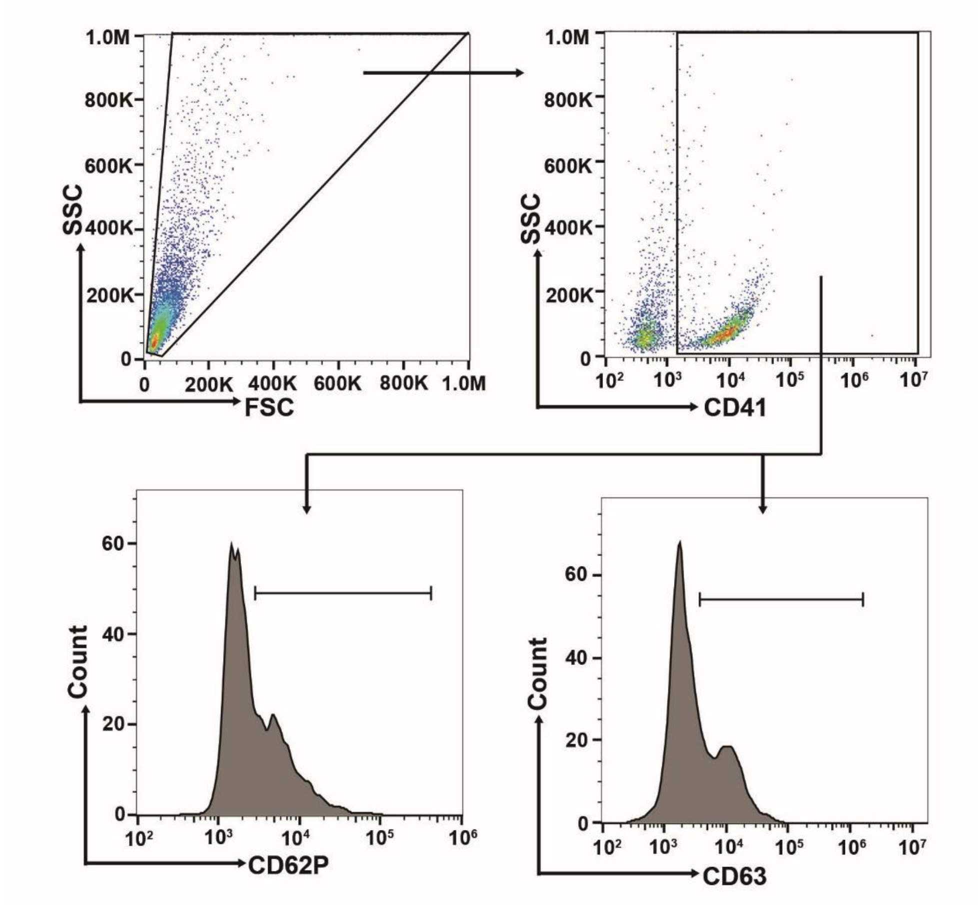


**Figure S10.** Gating strategy for identifying CD62P$^+$ and CD63$^+$ platelets. First, cells of the suitable size were selected on granularity (SSC) versus size (FSC) dot plots. The CD41$^+$ platelets were selected as show in the second granularity (SSC) versus CD41 dot plots. Finally, CD62P$^+$ or CD63$^+$ platelets were selected in the CD41$^+$ platelets based on negative samples and cell subsets.

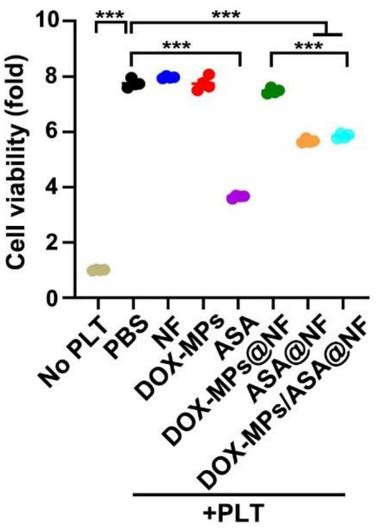


**Figure S11.** Cell viability of H22 cells at 48 h after incubation with or without PBS-, NF-, DOX-MPs-, ASA-, DOX-MPs@NF-, ASA@NF- or DOX-MPs/ASA@NF-pretreated platelets at the DOX concentration of 8 μg $mL^{-1}$ and ASA concentration of 600 μg $mL^{-1}$ for 12 h. Data are presented as mean ± s.e.m. (n = 4). ****P* < 0.001.

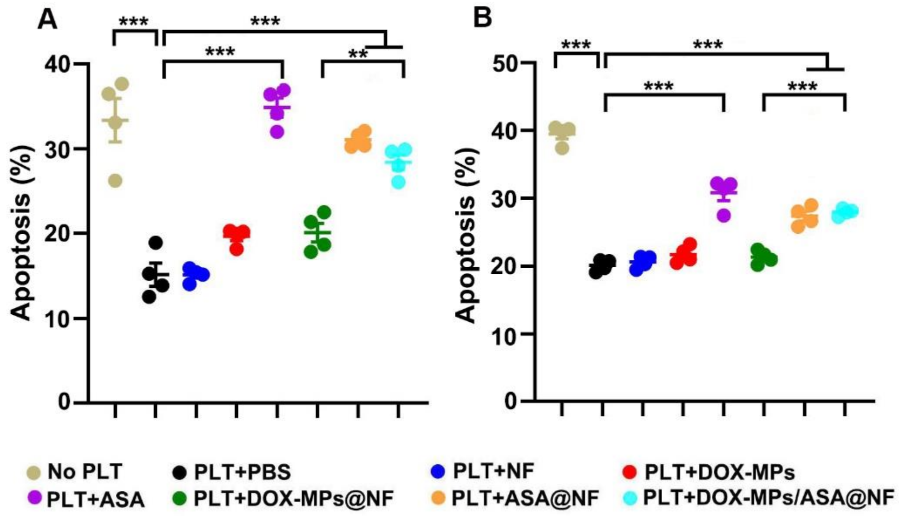


**Figure S12.** Effects of DOX-MPs/ASA@NF-pretreated platelets on the apoptosis of tumor cells in serum-free medium. (A,B) Apoptosis of 4T1 (A) and H22 (B) cells in serum-free medium at 72 h after incubation with or without PBS-, NF-, DOX-MPs-, ASA-, DOX-MPs@NF-, ASA@NF- or DOX-MPs/ASA@NF-pretreated platelets at the DOX concentration of 8 μg $mL^{-1}$ and ASA concentration of 600 μg $mL^{-1}$ for 12 h. Data are presented as mean ± s.e.m. (n = 4). **$P < 0.01$, ***$P < 0.001$.

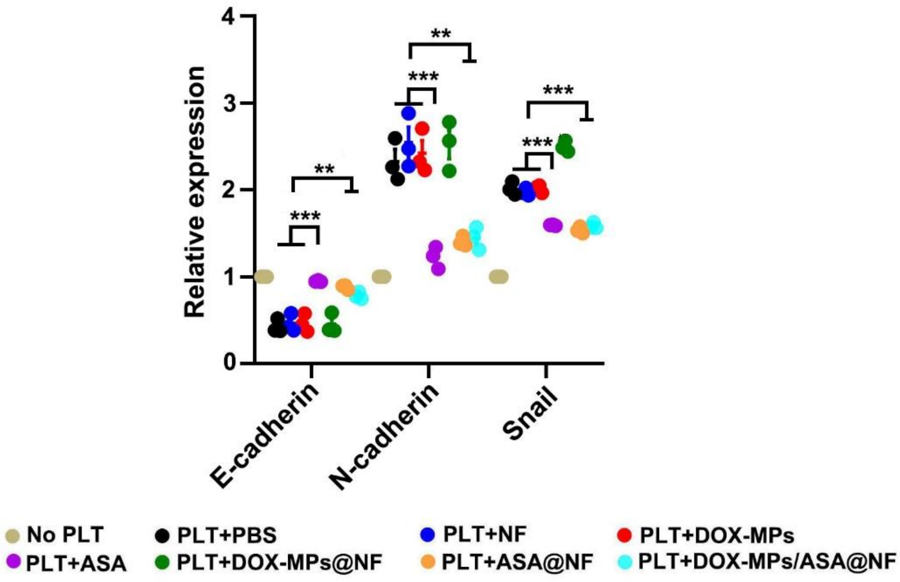


**Figure S13.** The quantification of E-cadherin, N-cadherin and Snail expression by western blot as shown in Figure 5c. Data are presented as mean ± s.e.m. (n = 3). $^{**}P < 0.01$, $^{***}P < 0.001$.

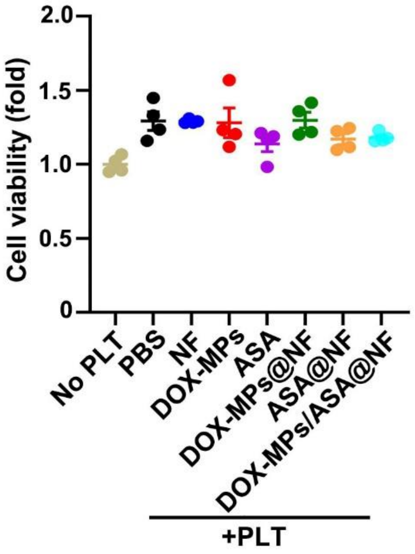


**Figure S14.** Cell viability of 4T1 cells at 18 h after incubation with or without PBS-, NF-, DOX-MPs-, ASA-, DOX-MPs@NF-, ASA@NF- or DOX-MPs/ASA@NF-pretreated platelets at the DOX concentration of 8 μg $mL^{-1}$ and ASA concentration of 600 μg $mL^{-1}$ for 12 h. Data are presented as mean ± s.e.m. (n = 4).

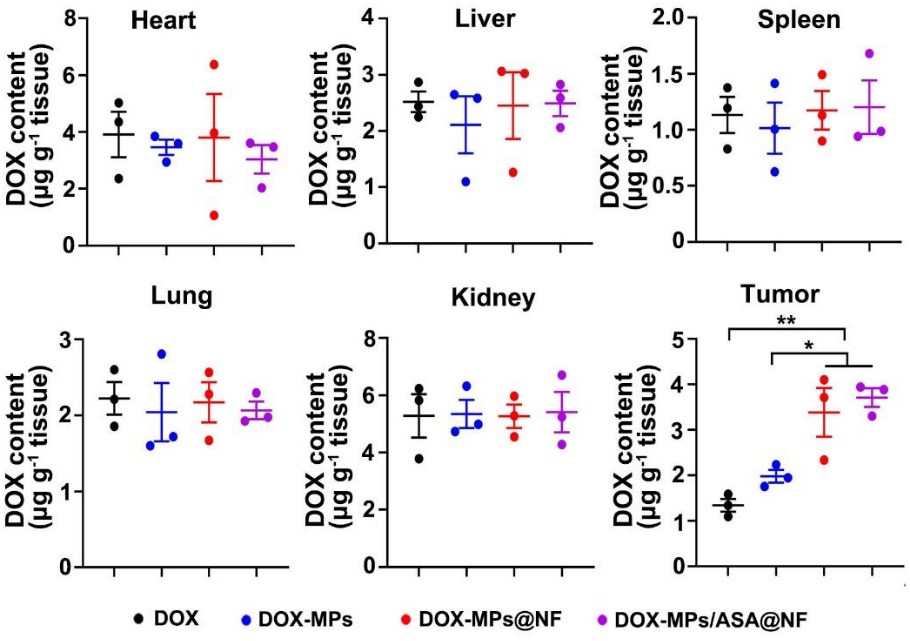


**Figure S15.** In vivo DOX biodistribution in 4T1 tumor-bearing mice undergoing surgical tumor resection at 72 h after implanting DOX, DOX-MPs, DOX-MPs@NF or DOX-MPs/ASA@NF at the DOX dosage of 1 mg $kg^{-1}$ and ASA dosage of 75 mg $kg^{-1}$ into the tumor resection cavity. Data are presented as mean ± s.e.m. ($n = 3$). *$P < 0.05$, **$P < 0.01$.

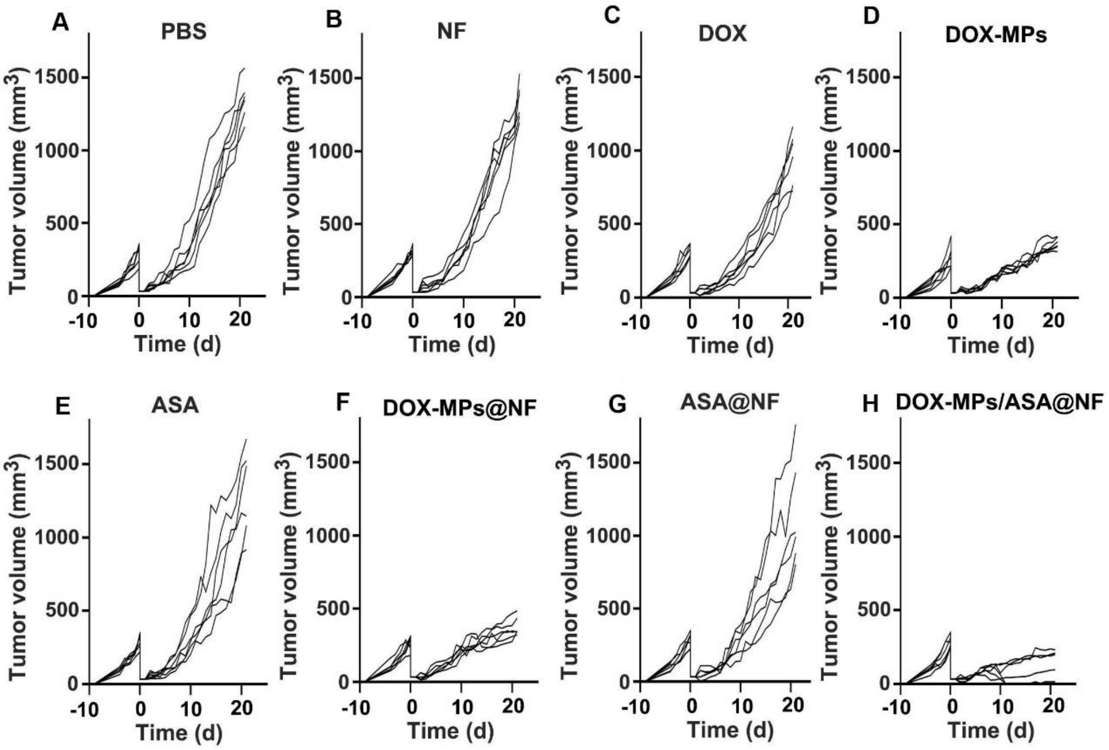


**Figure S16.** Anti-recurrence activity of DOX-MPs/ASA@NF in 4T1-Luc tumor-bearing mice after surgical tumor resection. (A-H) Individual tumor growth curves of 4T1-Luc tumor-bearing mice undergoing surgical tumor resection after implanting PBS (A), NF (B), DOX (C), DOX-MPs (D), ASA (E), DOX-MPs@NF (F), ASA@NF (G) or DOX-MPs/ASA@NF (H) at the DOX dosage of 1 mg $kg^{-1}$ and ASA dosage of 75 mg $kg^{-1}$ into the tumor resection cavity.

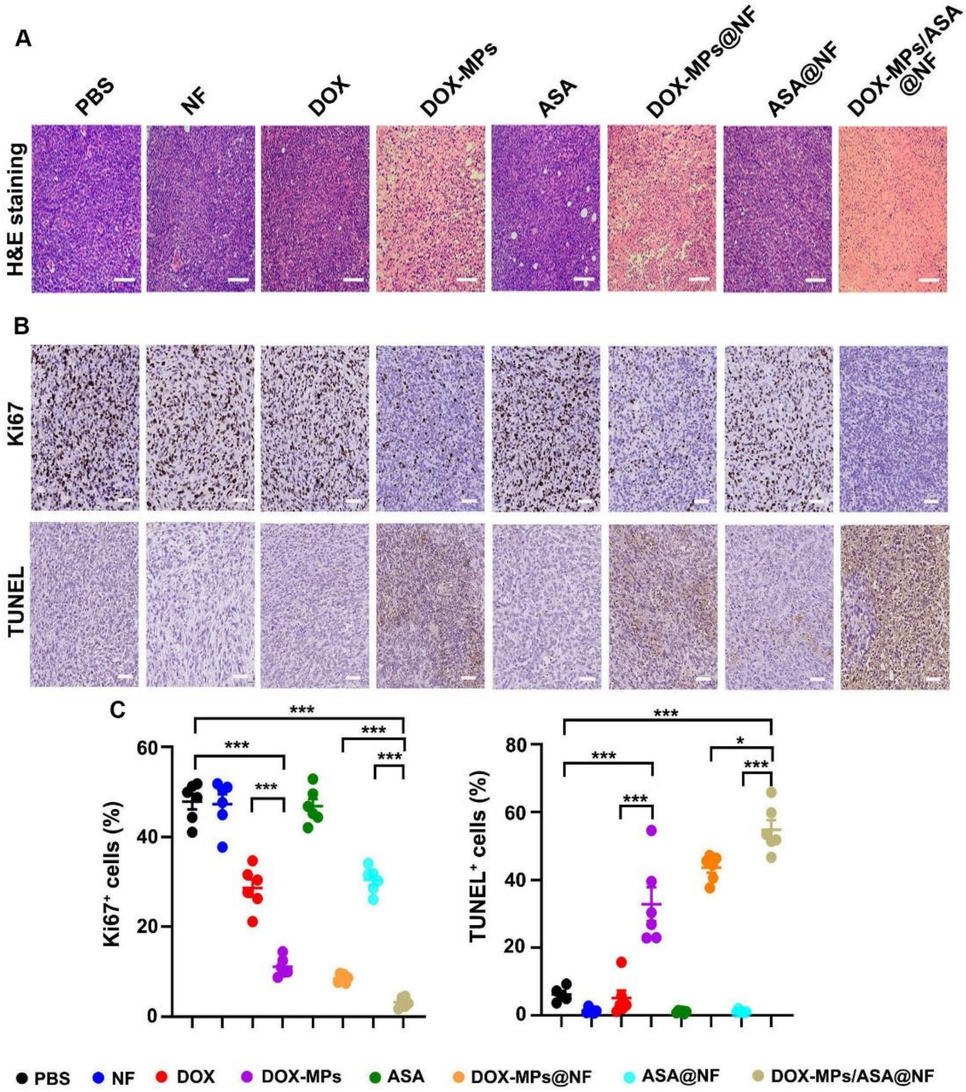


**Figure S17.** Efficient apoptosis induction and proliferation inhibition by DOX-MPs/ASA@NF in 4T1-Luc tumor-bearing mice undergoing surgical tumor resection. (A,B) Representative H&E (A), Ki67 and TUNEL staining (B) images of tumor sections in 4T1-Luc tumor-bearing mice undergoing surgical tumor resection at 25 days after implanting PBS, NF, DOX, DOX-MPs, ASA, DOX-MPs@NF, ASA@NF or DOX-MPs/ASA@NF at the DOX dosage of 1 mg $kg^{-1}$ and ASA dosage of 75 mg $kg^{-1}$ into the tumor resection cavity. Scale bars: 50 μm. (C) The quantification of $Ki67^{+}$ and $TUNEL^{+}$ cells in tumor tissues after treatment as above. Data are presented as mean ± s.e.m. (n = 6). **P*<0.05, ****P*<0.001.

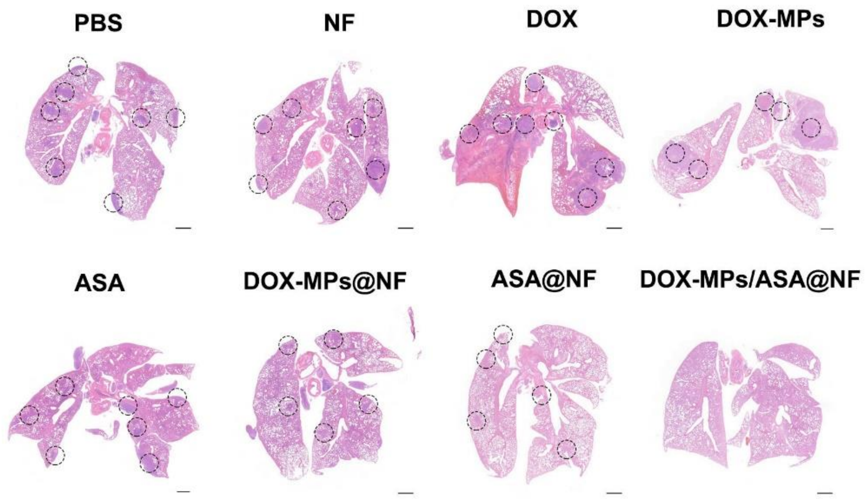


**Figure S18.** H&E staining of lung tissues of 4T1-Luc tumor-bearing mice undergoing surgical tumor resection at 25 days after implanting PBS, NF, DOX, DOX-MPs, ASA, DOX-MPs@NF, ASA@NF or DOX-MPs/ASA@NF at the DOX dosage of 1 mg $kg^{-1}$ and ASA dosage of 75 mg $kg^{-1}$ into the tumor resection cavity. The black circles indicated remarkable tumor nodules. Scale bars: 1 mm.

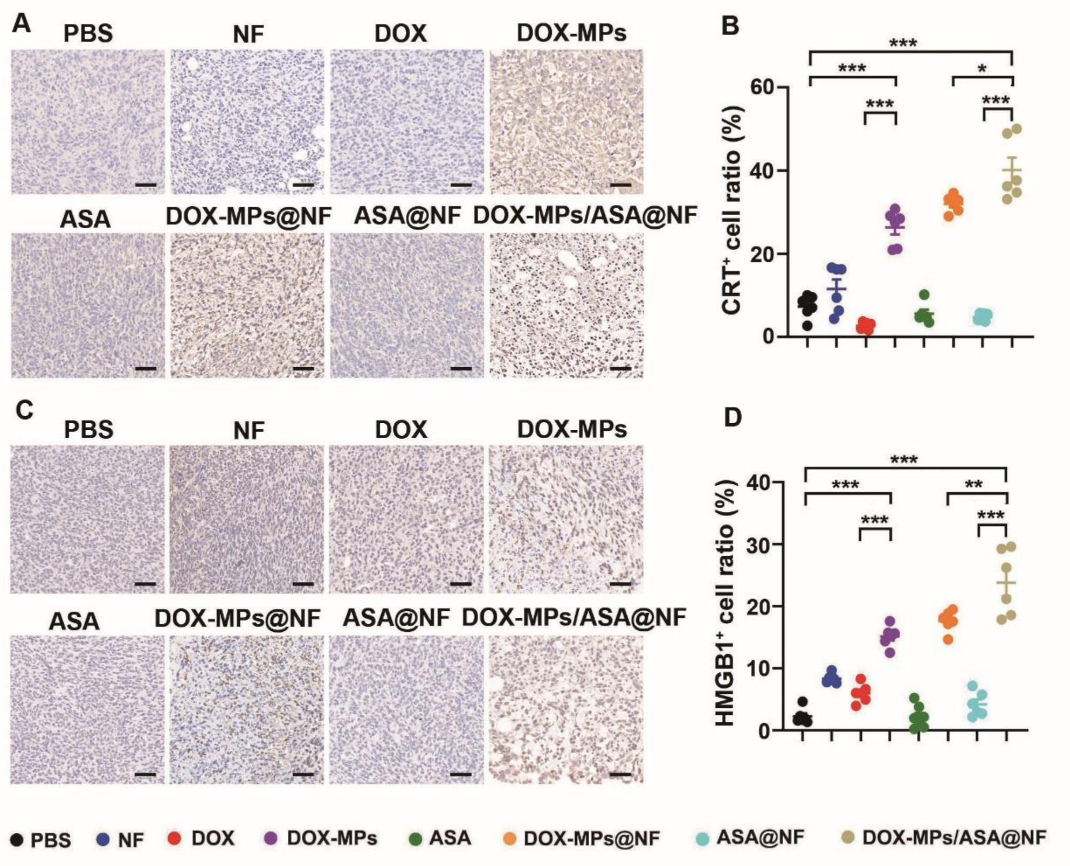


**Figure S19.** DOX-MPs/ASA@NF-induced ICD in tumor tissues of 4T1-Luc tumor-bearing mice undergoing surgical tumor resection. (A,C) Representative images of CRT (A) and HMGB1 (C) expression in tumors of 4T1-Luc tumor-bearing mice after implanting PBS, NF, DOX, DOX-MPs, ASA, DOX-MPs@NF, ASA@NF or DOX-MPs/ASA@NF at the DOX dosage of 1 mg $kg^{-1}$ and ASA dosage of 75 mg $kg^{-1}$ into the tumor resection cavity by immunohistochemistry. (B,D) The quantification of $CRT^{+}$ (B) and $HMGB1^{+}$ (D) cells in tumor tissues after treatment as above. Data are presented as mean ± s.e.m. (n = 6). *$P$<0.05, **$P$ < 0.01, ***$P$ < 0.001.

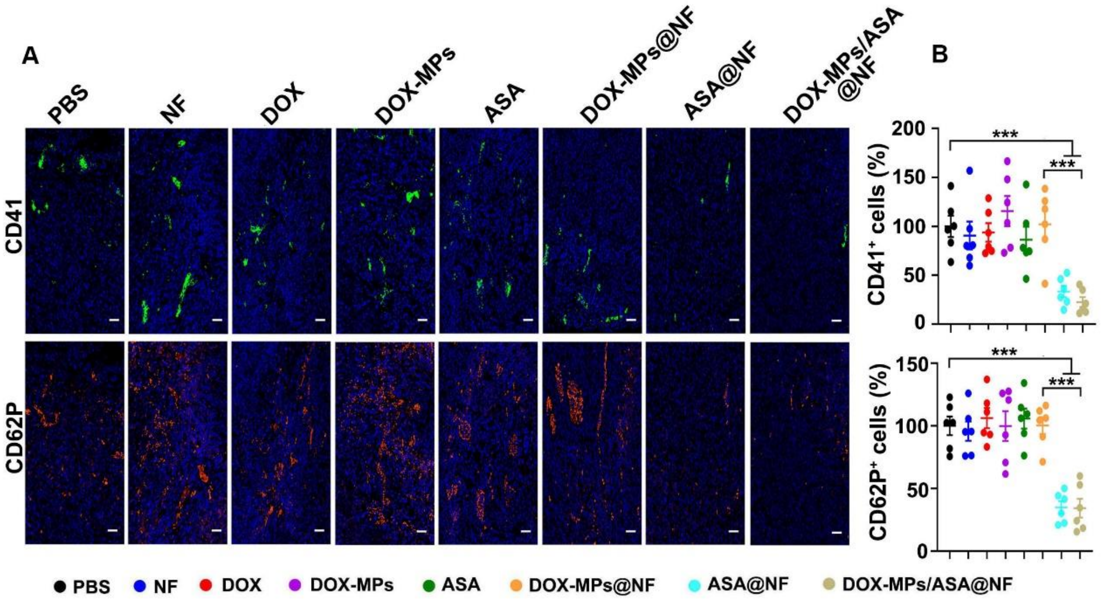


**Figure S20.** DOX-MPs/ASA@NF-induced inhibition of platelet activation in 4T1-Luc tumor-bearing mice undergoing surgical tumor resection. (A,B) Representative immunofluorescent staining images of CD41 and CD62P (A) and the quantification of $CD41^+$ and $CD62P^+$ cells (B) in tumor tissues of 4T1-Luc tumor-bearing mice undergoing surgical tumor resection at 25 days after implanting PBS, NF, DOX, DOX-MPs, ASA, DOX-MPs@NF, ASA@NF or DOX-MPs/ASA@NF at the DOX dosage of 1 mg $kg^{-1}$ and ASA dosage of 75 mg $kg^{-1}$ into the tumor resection cavity. Scale bars: 50 µm. Data are presented as mean ± s.e.m. (n = 6). ***$P < 0.001$.

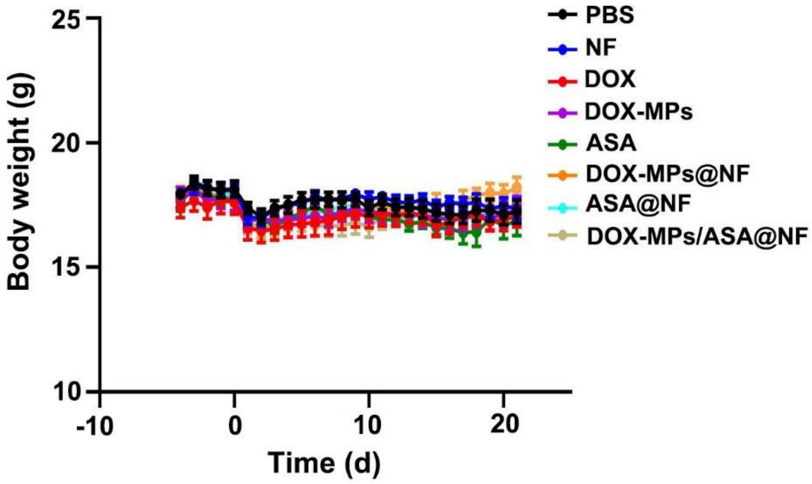


**Figure S21.** Body weight of 4T1-Luc tumor-bearing mice undergoing surgical tumor resection after implanting PBS, NF, DOX, DOX-MPs, ASA, DOX-MPs@NF, ASA@NF or DOX-MPs/ASA@NF at the DOX dosage of 1 mg $kg^{-1}$ and ASA dosage of 75 mg $kg^{-1}$ into the tumor resection cavity. Data are presented as mean ± s.e.m. (n = 6).

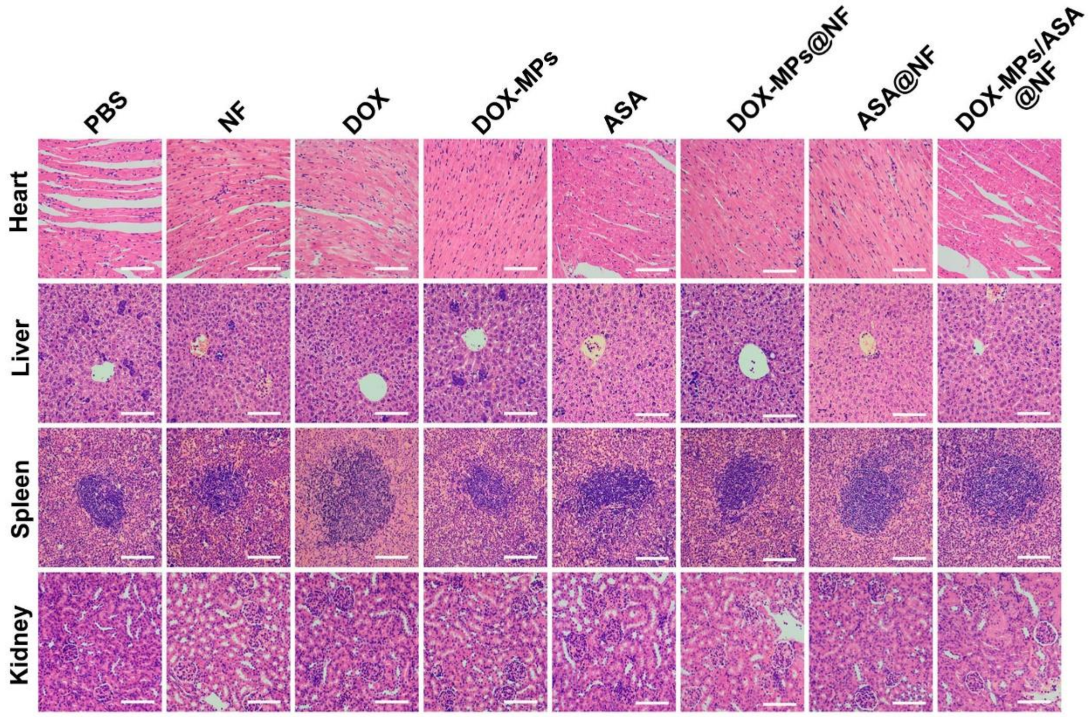


**Figure S22.** H&E staining of major organs of 4T1-Luc tumor-bearing mice undergoing surgical tumor resection at 25 days after implanting PBS, NF, DOX, DOX-MPs, ASA, DOX-MPs@NF, ASA@NF or DOX-MPs/ASA@NF at the DOX dosage of 1 mg $kg^{-1}$ and ASA dosage of 75 mg $kg^{-1}$ into the tumor resection cavity. Scale bars: 50 μm.

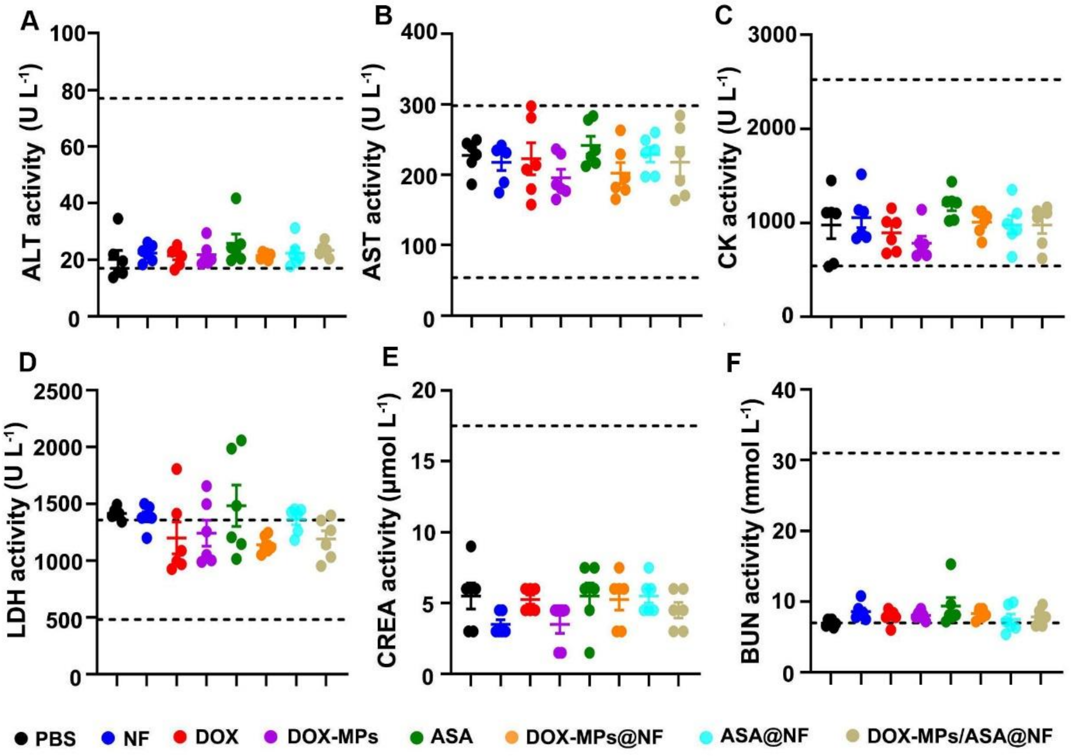


**Figure S23.** Serological analysis of 4T1-Luc tumor-bearing mice undergoing surgical tumor resection after treatment with DOX-MPs/ASA@NF. (A-F) The serological analysis of alanine aminotransferase (ALT, A), aspartate aminotransferase (AST, B), creatine kinase (CK, C), lactate dehydrogenase (LDH, D), creatinine (CREA, E) and blood urea nitrogen (BUN, F) in 4T1-Luc tumor-bearing mice undergoing surgical tumor resection at 25 days after implanting PBS, NF, DOX, DOX-MPs, ASA, DOX-MPs@NF, ASA@NF or DOX-MPs/ASA@NF at the DOX dosage of 1 mg $kg^{-1}$ and ASA dosage of 75 mg $kg^{-1}$ into the tumor resection cavity. Data are presented as mean ± s.e.m. (n = 6). The normal value range of serological analysis was shown within the horizontal lines.

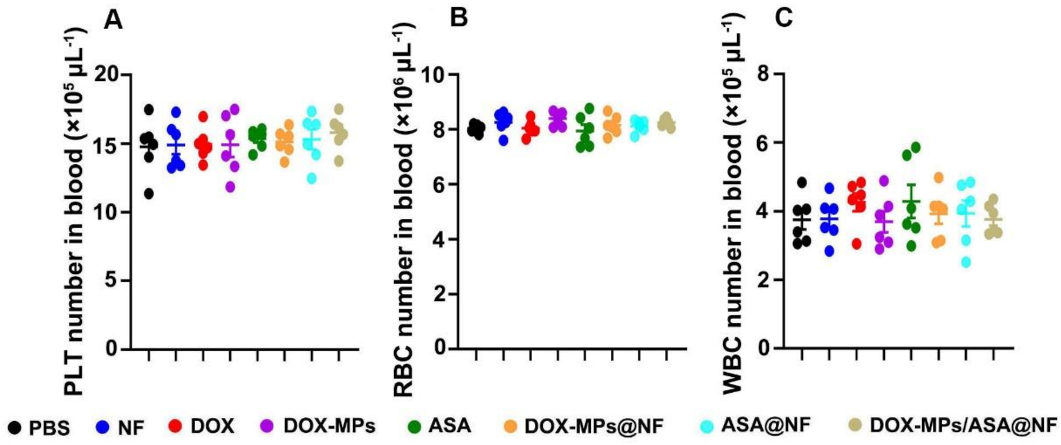


**Figure S24.** Effects of DOX-MPs/ASA@NF on the number of hemocytes in 4T1-Luc tumor-bearing mice undergoing surgical resection. (A-C) The number of platelets (A), red blood cells (RBCs, B) and white blood cells (WBCs, C) in the blood of 4T1-Luc tumor-bearing mice undergoing surgical resection at 25 days after implanting PBS, NF, DOX, DOX-MPs, ASA, DOX-MPs@NF, ASA@NF or DOX-MPs/ASA@NF at the DOX dosage of 1 mg $kg^{-1}$ and ASA dosage of 75 mg $kg^{-1}$ into the tumor resection cavity. Data are presented as mean ± s.e.m. (n = 6).

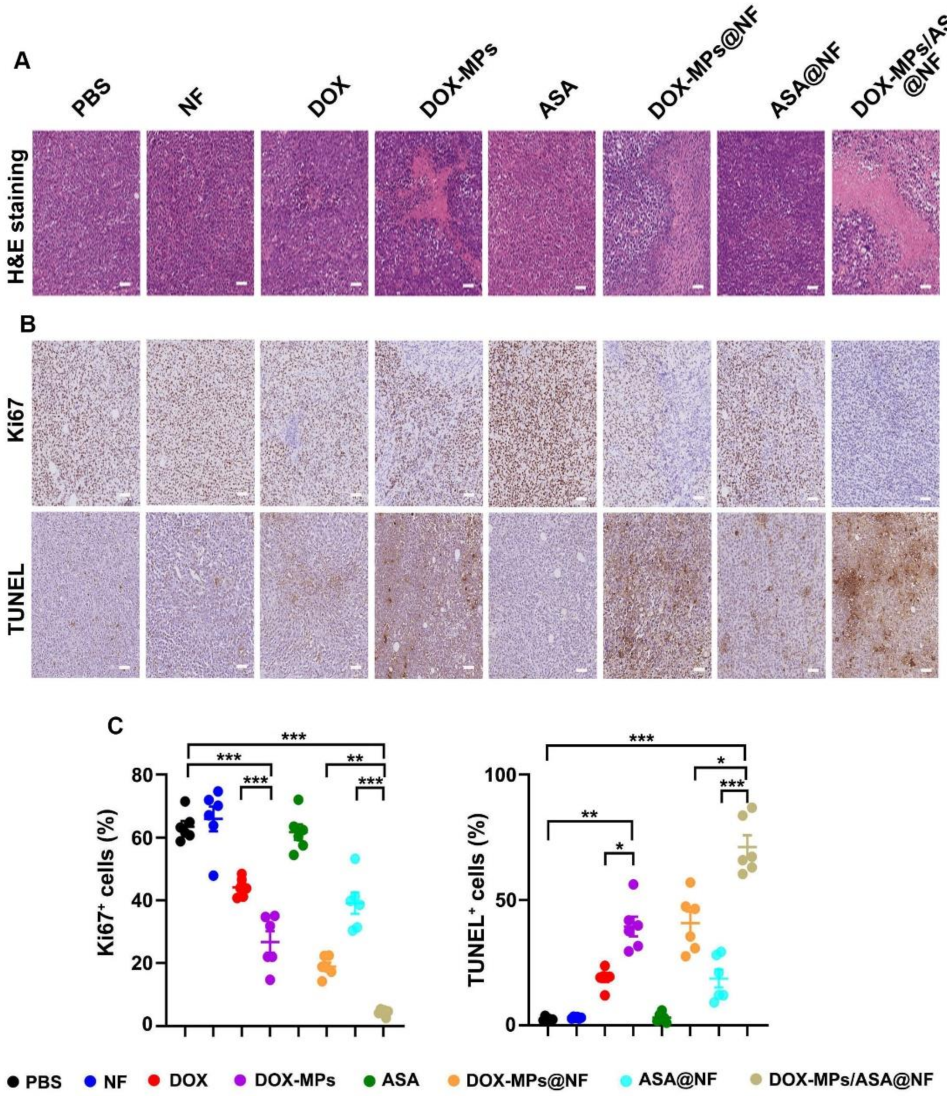


**Figure S25.** Efficient apoptosis induction and proliferation inhibition by DOX-MPs/ASA@NF in H22 tumor-bearing mice undergoing surgical tumor resection. (A,B) Representative H&E (A), Ki67 and TUNEL staining (B) images of tumor sections in H22 tumor-bearing mice undergoing surgical tumor resection at 24 days after implanting PBS, NF, DOX, DOX-MPs, ASA, DOX-MPs@NF, ASA@NF or DOX-MPs/ASA@NF at the DOX dosage of 1 mg $kg^{-1}$ and ASA dosage of 75 mg $kg^{-1}$ into the tumor resection cavity. Scale bars: 50 μm. (C) The quantification of $Ki67^{+}$ and $TUNEL^{+}$ cells in tumor tissues after treatment as above. Data are presented as mean ± s.e.m. (n=6). *$P$<0.05, **$P$<0.01, ***$P$<0.001.

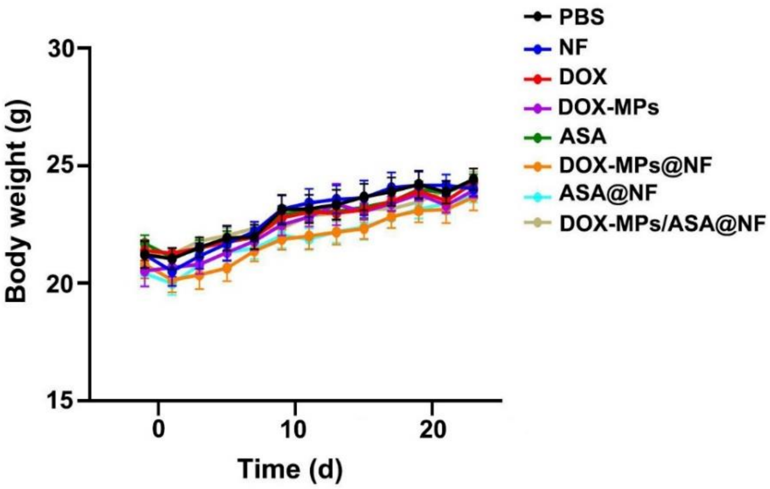


**Figure S26.** Body weight of H22 tumor-bearing mice undergoing surgical tumor resection after implanting PBS, NF, DOX, DOX-MPs, ASA, DOX-MPs@NF, ASA@NF or DOX-MPs/ASA@NF at the DOX dosage of 1 mg $kg^{-1}$ and ASA dosage of 75 mg $kg^{-1}$ into the tumor resection cavity. Data are presented as mean ± s.e.m (n = 6).

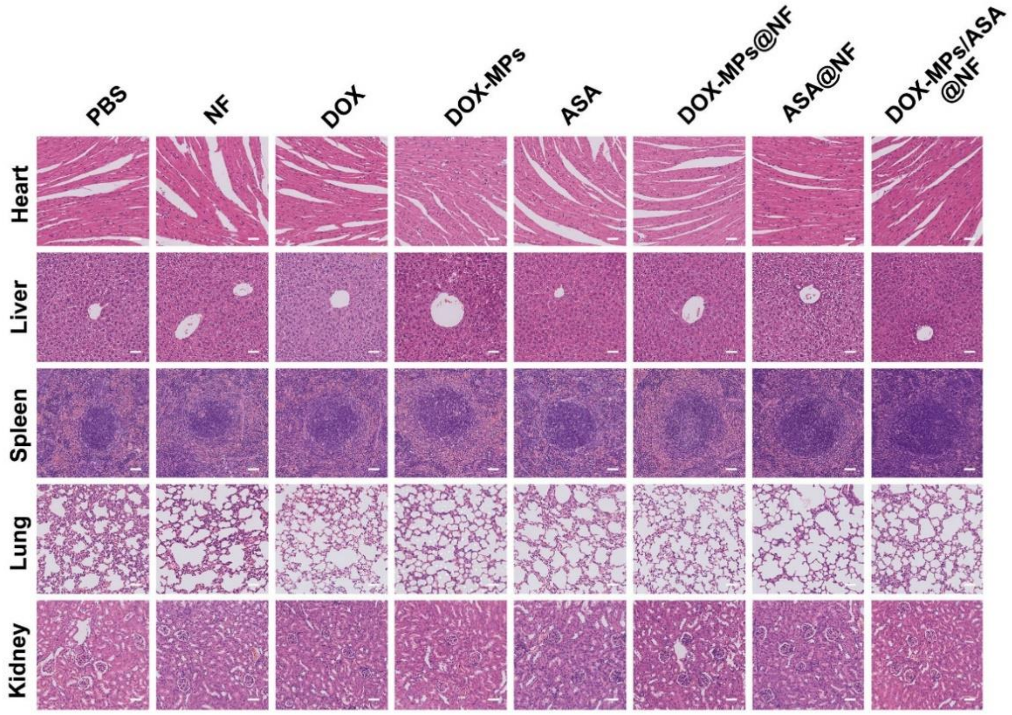


**Figure S27.** H&E staining of major organs of H22 tumor-bearing mice undergoing surgical tumor resection at 24 days after implanting PBS, NF, DOX, DOX-MPs, ASA, DOX-MPs@NF, ASA@NF or DOX-MPs/ASA@NF at the DOX dosage of 1 mg $kg^{-1}$ and ASA dosage of 75 mg $kg^{-1}$ into the tumor resection cavity. Scale bars: 50 μm.

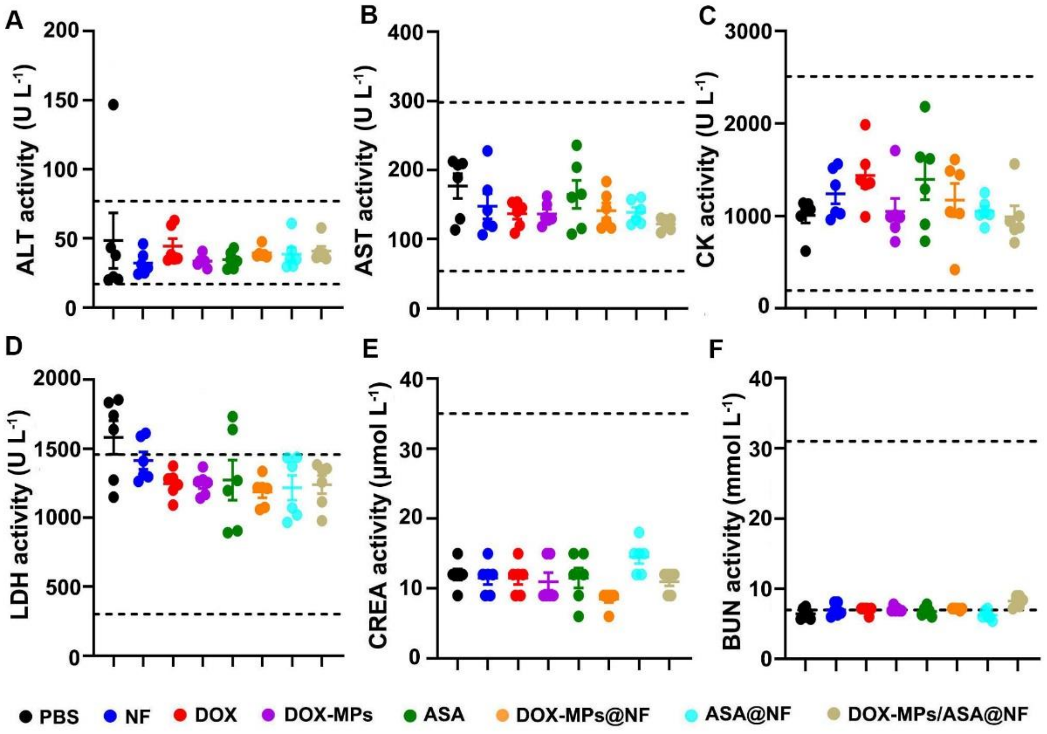


**Figure S28.** Serological analysis of H22 tumor-bearing mice undergoing surgical tumor resection after treatment with DOX-MPs/ASA@NF. (A-F) The serological analysis of ALT (A), AST (B), CK (C), LDH (D), CREA (E) and BUN (F) in H22 tumor-bearing mice undergoing surgical tumor resection at 24 days after implanting PBS, NF, DOX, DOX-MPs, ASA, DOX-MPs@NF, ASA@NF or DOX-MPs/ASA@NF at the DOX dosage of 1 mg $kg^{-1}$ and ASA dosage of 75 mg $kg^{-1}$ into the tumor resection cavity. Data are presented as mean ± s.e.m (n = 6). The normal value range of serological analysis was shown within the horizontal lines.

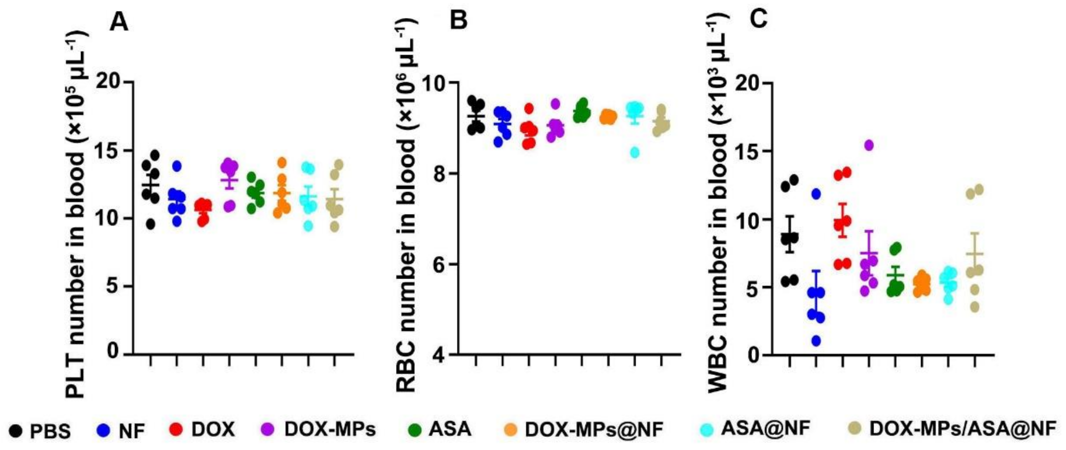


**Figure S29.** Effects of DOX-MPs/ASA@NF on the number of hemocytes in H22 tumor-bearing mice undergoing surgical resection. (A-C) The number of platelets (A), RBCs (B) and WBCs (C) in the blood of H22 tumor-bearing mice undergoing surgical resection at 24 days after implanting PBS, NF, DOX, DOX-MPs, ASA, DOX-MPs@NF, ASA@NF or DOX-MPs/ASA@NF at the DOX dosage of 1 mg $kg^{-1}$ and ASA dosage of 75 mg $kg^{-1}$ into the tumor resection cavity. Data are presented as mean ± s.e.m. (n = 6).
